# Hexavalent Chromium Induces Apoptosis and Autophagy in Human Neurons and Astrocytes via MAPK Pathway Activation

**DOI:** 10.1007/s12011-026-05046-0

**Published:** 2026-04-05

**Authors:** Suttinee Phuagkhaopong, Ratchanon Sukprasert, Rapeewan Settacomkul, Dusadee Ospondpant, Kran Suknuntha, Phisit Khemawoot, Christopher Power, Pornpun Vivithanaporn

**Affiliations:** 1 Department of Pharmacology, Faculty of Medicine, Chulalongkorn University, Bangkok, Thailand; 2 Chakri Naruebodindra Medical Institute, Faculty of Medicine Ramathibodi Hospital , Mahidol University, Samut Prakan, 10540 Thailand; 3 Department of Medicine, University of Alberta, Edmonton, AB Canada; 4 Ramathibodi Medical School, Faculty of Medicine Ramathibodi Hospital, Mahidol University, Samut Prakan, Thailand

**Keywords:** Chromium (VI), Apoptosis, Autophagy, Antiproliferation, MAPK signalling pathway

## Abstract

Hexavalent chromium (Cr[VI]), one of the major heavy metals in fine particulate matter (PM_2.5_), has been linked to increased risks of neurological impairment and brain cancer. However, the direct effect of Cr(VI) on brain cells remains unclear. In this study, we investigated the cell type–specific cytotoxicity of Cr(VI) at environmentally relevant concentrations to cultured human neurons and astrocytes. Our findings showed that higher concentrations of Cr(VI) were required to induce cell death in human astrocytes (IC_50_ of 32.05 µM at 24 h) than in the human neurons (IC_50_ of 24.55 µM at 24 h). The neurotoxicity effects associated with elevated intracellular chromium levels included DNA damage (decreased poly[ADP–ribose] polymerase expression), mitochondria-mediated apoptosis (increased caspase 3/7 activation), and autophagy (increased light chain 3 [LC3]–II to LC3–I levels accompanied by decreased p62 expression). Activation of the MAPK signalling pathway triggered Cr(VI) –induced brain death. Furthermore, Cr(VI) exposure induced S–phase accumulation in U-87 MG cells by altering cell cycle–related protein expression, specifically by upregulating CDK inhibitors (p21, p27, and p53) and downregulating cyclin B, cyclin D, and CDK4. Together, these findings demonstrate that the mechanisms underlying Cr(VI)-induced neurotoxicity were similar between the two cell types and further highlight the importance of increased awareness of chromium pollution and its impact on human health.

**Graphical Abstract:**

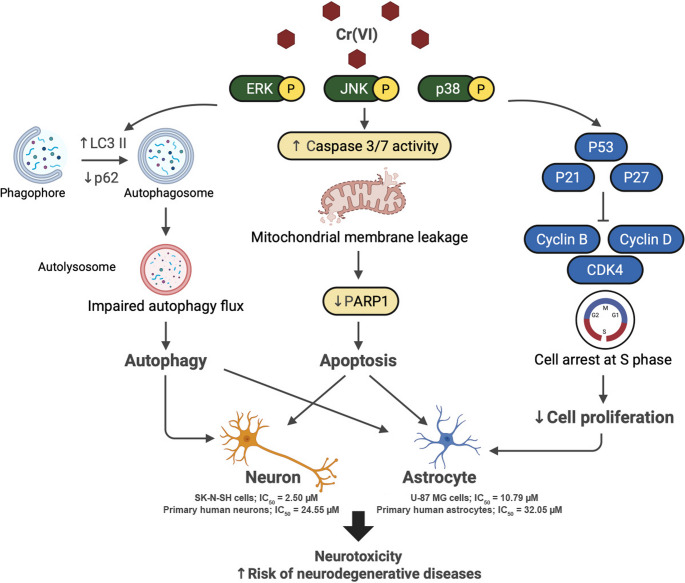

**Supplementary Information:**

The online version contains supplementary material available at 10.1007/s12011-026-05046-0.

## Introduction

Hexavalent chromium (Cr[VI]) is a global environmental pollutant classified as a group 1 carcinogen by the International Agency for Research on Cancer (IARC) and the US Environmental Protection Agency (US EPA), with primary associations to lung cancer [1]. Chromium is primarily produced through mining and industrial processes. Exposure to chromium through the inhalation of airborne particles is the main cause of chromium poisoning. Ingestion of 5 g of chromic acid is considered acute lethal condition in humans [2]. Studies in chromium-contaminated residual areas indicate a relationship between chromium exposure levels and adverse human health impacts, particularly stomach, lung, kidney, and general urinary cancer, dermatological disorders, and hematological disorders [3]. Villagers in China living in areas near ferrochromium factories with Cr(VI) contamination in drinking water exhibit a higher risk of stomach cancer (RR = 1.82; 95% CI = 1.11–2.91) and a slightly elevated risk of lung cancer (RR = 1.15; 95% CI = 0.62–2.07) compared with villagers living in non-contaminated areas [4]. The presence of Cr(VI) in groundwater at concentrations exceeding 20 ppm (~ 67.1 µM sodium dichromate dihydrate), which is approximately 390 times higher than the threshold limit value recommended by the World health organization (0.05 ppm; 0.2 µM) caused gastrointestinal issues and hematological abnormalities in exposed Indian residents [5]. In addition, workers at chromate production facility generating airborne Cr(VI) exhibited significantly higher blood chromium levels (median = 15.68 ppb; 0.05 µM) compared with unexposed healthy workers (median = 3.03 ppb; 0.01 µM), which was associated with adverse effects on blood element homeostasis [6].

Chromium exposure is associated with an increased risk for neurological complications and brain cancer [7, 8]. Chromium persists five times longer in brain tissue, with a half-life of 2.82 days versus 0.66 days in lung tissue [9]. Exposure to chromium leads to detrimental neuropsychological development in children living in a coastal industrialized region in Spain and motor neuron impairment in people residing in areas with high chromium contamination [10, 11]. Intranasal exposure to chromium in rats induces brain injury by penetrating the nasal membrane and entering the brain via the olfactory pathway [12]. Among brain cells, astrocytes are the most abundant and possess unique protecting mechanisms against toxicity caused by heavy metals [13]. Chronic intranasal exposure to chromium in rats increases the number of activated astrocytes (astrogliosis), resulting in an accelerated decrease in survival of neurons [14]. Studies have shown that chromium induces neurotoxicity by interfering with mitochondrial functions and inducing DNA damage in human neuroblastoma [15] and rat primary neurons [16]. Although evidence has emerged regarding the influence of astrocyte function and survival on neurons [17], comparative data on the cytotoxicity of chromium on neurons and astrocytes remain scarce.

Several studies have suggested that Cr(VI) toxicity induced cytotoxicity mainly via MAPK pathway, a pivotal signalling cascade activated in response to immediate cellular stress, in human lung epithelial cells and also in murine models [18]. However, it is not well understood whether the MAPK pathway is activated in brain cells following Cr(VI) exposure. Therefore, we aim to investigate the cell type–specific responses associated with the cytotoxic effects of the exposure of human neurons and astrocytes to Cr(VI) by examining the underlying molecular mechanisms. The identification of pathways through which Cr(VI) induces neurotoxicity as achieved here should deepen our understanding of heavy metal–induced neurological dysfunctions and thus pave the way for the development of effective diagnostic and therapeutic strategies.

## Materials and Methods

### Cell Cultures

A human neuronal cell line (SK-N-SH) and a human astrocyte cell line (U-87 MG) were obtained from the American Type Culture Collection (ATCC, USA). The culture medium for SK-N-SH and U-87 MG cells were composed of Eagle’s Minimum Essential Medium (MEM; Gibco, USA), 10% (v/v) foetal bovine serum (FBS; Gibco, USA), 100 U/mL penicillin, and 100 µg/mL streptomycin, with 1mM sodium pyruvate (Gibco, USA) added for U-87 MG. The medium was changed every 1–2 days.

A primary human neurons (PHN) and a primary human astrocytes (PHA) were obtained from foetuses aborted at 15–20 weeks of gestation with written consent from donors under protocol 27,660 approved by the University of Alberta Human Research Ethics Board (Biomedical) and were prepared as reported previously [19]. Briefly, the aborted fetuses were dissected to remove meninges and were digested with 2.5% trypsin and 0.2 mg/mL DNase I for 60 min. After that, the digested tissues were filtered through 70 μm cell strainers to remove undigested tissue and cell clumps and were washed twice with fresh medium. The culture medium for PHN was composed of Neurobasal medium (Gibco, USA), 1X B-27, 0.25X GlutaMAX, 29.7 nM cytosine arabinoside, 100 U/mL penicillin, and 100 µg/mL streptomycin (Gibco, USA). The cells were maintained for 14 days prior to treatment, with half of the medium replaced every 3 days. The culture medium for PHA was composed of MEM (Gibco, USA), 10% (v/v) FBS, 2 mM L–glutamine, 1 mM sodium pyruvate, 1X MEM non-essential amino acids, 0.1% dextrose, 0.5 µg/mL amphotericin B, and 20 µg/mL gentamicin, 100 U/mL penicillin, and 100 µg/mL streptomycin (Gibco, USA). PHA were passaged 3–4 times to achieve purity and maturity before use.

## Preparation of Sodium Dichromate and Reagents

Sodium dichromate dihydrate (Na_2_Cr_2_O_7_-2H_2_O; Molecular Weight, 298.00; Sigma, USA) was dissolved in sterile water at a concentration of 100 mM and stored at −20 °C. Chromium was freshly thawed and diluted in MEM to the indicated concentrations before use. U0126 (MEK1/2 inhibitor; Cell-signaling, USA), SB203580 (p38 inhibitor; Tocris, United Kingdom), SP600125 (JNK inhibitor; Sigma-Aldrich, USA) were dissolved in dimethyl sulfoxide (DMSO; Sigma, USA) and stored at −20 °C. Cells were co−treated with Cr(VI) and inhibitors, with final DMSO concentration of 0.1% (v/v).

## MTT Cell Viability Assay

Viability of the cultured cells was determined by 3−(4,5−dimethylthiazol−2−yl)−2,5−diphenyltetrazolium bromide (MTT) assays. Specifically, the cells were placed in 96−well culture plates at density of 22,000 cell/well for SK-N-SH and 20,000 cell/well for U-87 MG. The cells were incubated with Cr(VI) at different concentrations in the range of 1nM to 1,000 µM for 24 and 48 h. MTT (Bioline Corporation, Canada) was dissolved in phosphate-buffered saline (PBS), and the MTT solution was added to each well to a final concentration of 0.5 mg/mL for 4 h at 37 °C. Formazan crystals were then dissolved in DMSO and measured spectrophotometrically at 562 nm with background subtraction at 690 nm using a microplate reader (Cytation 5 Imaging Reader; Biotek, USA). Cell viability was calculated as a percentage relative to that of untreated cells.

## PrestoBlue Cell Viability Assay

Viability of the cultured cells was determined by resazurin−based PrestoBlue assay. Specifically, the cells were placed in 96−well culture plates at density of 22,000 cell/well for SK-N-SH, 20,000 cell/well for U-87 MG, 50,000 cells/well for PHN and 18,000 cells/well for PHA. The cells were incubated with Cr(VI) at different concentrations in the range of 1nM to 1,000 µM for 24 and 48 h. PrestoBlue reagent (A13261, Invitrogen) was added to each well to a final concentration of 10% (v/v) for 4 h at 37 °C and were measured at 570 nm with background subtraction at 600 nm using a Victor Nivo multimode plate reader (PerkinElmer, Australia). Cell viability was calculated as a percentage relative to that of untreated cells.

## Lactate Dehydrogenase Cell Cytotoxicity Assay

Cytotoxicity effect of Cr(VI) in PHN and PHA was determined by measuring the lactate dehydrogenase (LDH) released from cells. Specifically, the cells were placed in 96−well culture plates at density of 50,000 cells/well for PHN and 18,000 cells/well for PHA and were incubated with Cr(VI) at concentrations of 1, 10, and 100 µM for 24 and 48 h. After the treatment, the culture medium of the treated cells was collected and assessed for LDH activity using LDH assay kits according to the manufacturer’s instruction (Ab65393, Abcam). Absorbance was measured were measured at 450 nm with background subtraction at 650 nm using a Victor Nivo multimode plate reader (PerkinElmer). Cell viability was calculated as a percentage relative to that of untreated cells.

### Measurement of Intracellular Chromium

To measure intracellular chromium, 1.5 × 10^6^ cells were placed in 100 mm culture dishes. At 70%–80% confluence, the cells were treated with Cr(VI) at concentrations of 1, 2.5, 5, and 10 µM for 6 and 24 h. The cells were then washed twice with PBS containing 10 mM disodium ethylenediamine tetraacetic acid (EDTA) and twice with PBS without EDTA. They were then trypsinised with 0.1% trypsin and centrifuged at 3,000 x g for 10 min. Cell pellets were weighed as wet weight, digested with 1 mL of 65% (v/v) nitric acid, and evaporated at 90 °C three times. The lysate was incubated with 2 mL of 65% (v/v) nitric acid overnight and then diluted with 18 mL of distilled water. Cr(VI) content was measured in mg/L or ppm using a flame furnace atomic absorption spectrophotometer (PinAAcle 900T; PerkinElmer, Massachusetts, USA). The intracellular Cr(VI) content was expressed as µg/g (Cr(VI)/protein wet weight).

## Flow Cytometric Analysis of Cell Death

To measure cell death, 2 × 10^6^ cells were placed in 6−well culture plates and exposed to Cr(VI) for 24 h. Then, the cells were trypsinised, resuspended in 50 µL of cold 1⋅ binding buffer, and stained with 2 µL of Annexin V–FITC and 5 µL of 7−aminoactinomycin D (7−AAD) solution, in accordance with the manufacturer’s instructions (BD Biosciences, USA). After staining, the mixture was incubated in the dark at room temperature for 15 min. After incubation, an additional 200 µL of 1⋅ binding buffer was added. A total of 10,000 events were immediately analysed per sample using a BD Accuri C6 Plus flow cytometer (BD Biosciences, USA). Cell death was determined after double staining of the cells with Annexin V–FITC and 7−AAD, which allowed determination of the proportions of viable cells (Annexin V^−^/7−AAD^−^), early apoptotic cells (Annexin V^+^/7−AAD^−^), necrotic cells (Annexin V^−^/7−AAD^+^), and late apoptotic cells (Annexin V^+^/7−AAD^+^).

## Flow Cytometric Analysis of Cell Proliferation

To measure cell proliferation, 5 × 10^5^ cells were placed in 6−well culture plates containing MEM supplemented with 5% (v/v) FBS after staining with 1 µM carboxyfluorescein N−succinimidyl ester (CFSE) (Sigma, USA), a green−fluorescent dye that permeates the cell membrane and covalently binds to cytosolic components. Beginning the next day, the cells were treated with Cr(VI) at concentrations of 0.1 and 1 µM in MEM with 5% (v/v) FBS for 48 h. After each cell division, the dye was equally distributed into two daughter cells; thus, the daughter cells exhibited half the fluorescence intensity of their parent cells. Following incubation, the cells were harvested, and a total of 10,000 events were immediately analysed per sample. Mean fluorescence was recorded at FL1 peak emission (wavelength 517 nm) using a BD Accuri C6 Plus flow cytometer (BD Biosciences, USA).

### Flow Cytometric Analysis of Cell Cycle Distribution

To measure the cell cycle distribution, 1 × 10^6^ cells were placed in 6−well culture plates. After 24 h of starvation, the cells were treated with 0.1 and 1 µM Cr(VI) for 24 h. Then, the cells were labelled with 10 µM bromodeoxyuridine (BrdU) for 1 h and then detached with 0.1% (v/v) trypsin–EDTA at 37 °C for 3 min. Next, the cells were fixed, permeabilised, and stained with FITC–conjugated anti–BrdU antibody and 7–AAD (BD Pharmingen, BD Biosciences, USA), in accordance with the manufacturer’s protocol. The number of cells in each phase of the cell cycle was quantified using a BD Accuri C6 Plus flow cytometer (BD Biosciences, USA), and the proportions of cells in the sub–G_1_, G_1_, S, and G_2_/M phases of the cell cycle were determined using BD Accuri C6 Plus software (BD Biosciences, USA). A minimum of 10,000 events were analysed for each sample.

### Flow Cytometric Analysis of Autophagy

Following Cr(VI) exposure, cells were stained with a CYTO-ID^®^ Green autophagy detection kit (Enzo Life Science, USA), in accordance with the manufacturer’s protocol. Briefly, the cells were washed twice with PBS and stained with CYTO-ID^®^ Green dye diluted in 1⋅ assay buffer (1:1000) at 37 °C for 30 min. Next, the cells were again washed twice with assay buffer and supplemented with an additional 500 µL of 1⋅ assay buffer. A total of 10,000 events were immediately analysed per sample. Mean fluorescence intensity (MFI) was recorded at FL1 peak emission (wavelength 480 nm) using a BD Accuri C6 Plus flow cytometer (BD Biosciences, USA).

### Western Blot Analysis

Following exposure to Cr(VI), cells were treated with lysis buffer (10 mM HEPES; pH 7.5, 10 mM KCl, 0.1 mM EDTA, 1 mM dithiothreitol [DTT], 0.5% Nonidet P–40, and 0.5 mM phenylmethylsulfonyl fluoride along with the protease inhibitor cocktail) and allowed to swell on ice for 15–20 min with intermittent mixing. Tubes were vortexed to disrupt cell membranes and then centrifuged at 12,000 x g and 4 °C for 10 min. The concentrations of the proteins were determined using Bradford protein assays (Bio-Rad, USA). Loading buffer (0.125 M Tris-HCl, pH 6.8, 2% [v/v] sodium dodecyl sulphate [SDS], 0.5% [v/v] 2-mercaptoethanol, 1% [v/v] bromophenol blue, and 20% [v/v] glycerol) was added to the protein sample and heated for 5 min at 95 °C. Ten micrograms of protein samples were separated on 12% (v/v) SDS/polyacrylamide gels and transferred to nitrocellulose membranes. These membranes were blocked with 5% (w/v) non-fat milk in Tris-buffered saline containing 0.1% (v/v) Tween-20 (TBS-T) for 1 h and incubated with specific primary antibodies for a mitochondrial apoptotic marker (cleaved-caspase 3), a DNA damage marker (PARP1), and cell cycle–related proteins (cyclin A, cyclin B, cyclin D, CDK2, CDK4, p21, p27, and p53) overnight at 4 °C. Membranes were washed three times with wash buffer TBS-T and incubated with a secondary anti-rabbit IgG or anti-mouse IgG (Cell Signalling Technologies, Danvers, MA, USA) antibody conjugated to the enzyme horseradish peroxidase (HRP) for 1 h. Membranes were washed three times and proteins were detected using the enhanced chemiluminescence method as specified by the manufacturer. Autoradiography signals were assessed using a digital imaging system (ImageQuant™ 800; Cytiva Life Sciences, USA). Band density was determined using ImageJ software. β-Actin was used as an internal loading control to normalise the expression of proteins of interest.

### IncuCyte Live Cell Imaging

For live cell imaging, 2 × 10^4^ cells were placed in 96–well clear–bottom culture plates overnight, after which they were treated with Cr(VI) at concentrations of 0.1 and 1 µM in MEM supplemented with caspase 3/7 red dye (#4704; Sartorius). Images were captured using an IncuCyte live cell imaging system (IncuCyte S3; Sartorius; humidified, 5% CO_2_, 37 °C) every 3 h up to 72 h. The mean fluorescence intensity (MFI) for caspase 3/7 red dye was calculated using IncuCyte^®^ S3 live cell analysis system software.

### Statistical Analysis

All data are presented as mean ± standard error of mean (SEM). Data analysis was performed using GraphPad Prism version 10 (GraphPad Software, San Diego, CA, USA; www.graphpad.com). The statistical significance of differences between mean values was evaluated by analysis of variance (ANOVA) followed by Tukey’s post hoc test for multiple-group comparisons or independent sample t-test. In all comparisons, differences were considered significant when P values were less than 0.05. The number of independent biological experiments (n) is noted in each figure legend.

## Results

### Cr(VI) Exposure Reduced Cell Viability More in Neurons than in Astrocytes

**Fig. 1:**
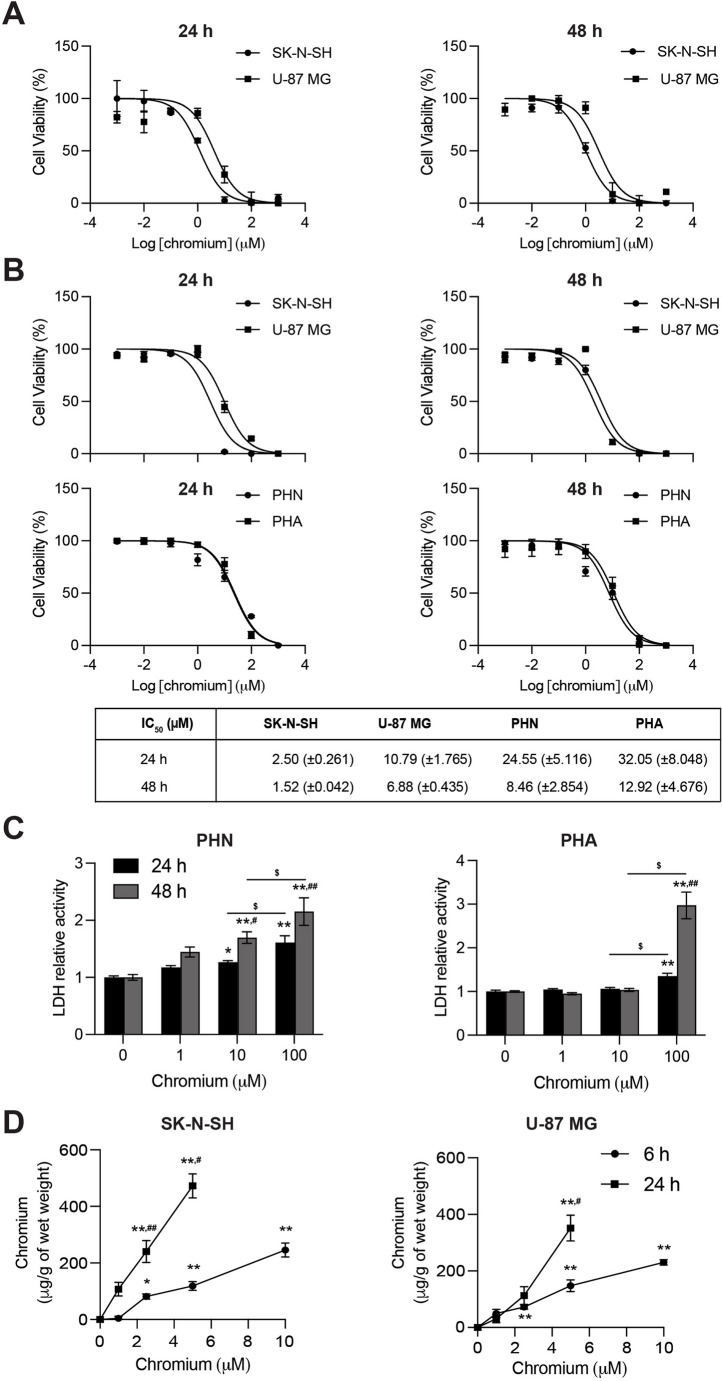
Cytotoxicity of Cr(VI) in human neurons and human astrocytes. **A** Viability of human neuronal (SK-N-SH) and human astrocyte (U-87 MG) cell lines was determined using MTT assays following exposure to Cr(VI) at 0.1–1000 µM for 24 and 48 h (*n* = 5). Cr(VI) reduced the numbers of both cell types in a concentration-dependent manner. **B** Viability of SK-N-SH, U-87 MG, primary human neurons (PHN), and primary human astrocytes (PHA) was determined using PrestoBlue assays following exposure to Cr(VI) at 0.1–1000 µM for 24 and 48 h (*n* = 5). Cr(VI) reduced the viability of primary neurons more than that of primary human astrocytes. **C** Activity of lactate dehydrogenase was measured in the culture medium of Cr(VI) –treated cells (*n* = 5). Cr(VI) showed cytotoxic effects to primary neurons and primary astrocytes as evidenced by PrestoBlue assays. **D** Accumulation of Cr(VI) in SK-N-SH and U-87 MG cells was detected by flame atomic absorption spectrometry following exposure to Cr(VI) at 1–10 µM for 6 and 24 h (*n* = 4). Cr(VI) accumulated in both cell types in a concentration- and time-dependent manner. All data are presented as mean ± SEM. Statistically significant differences are shown as *p < 0.05 and **p < 0.01 (when comparing mock-treated cells at the same timepoint), ^#^*p* < 0.05 when comparing chromium-treated cells at the same concentration but different timepoints, and ^$^*p* < 0.05 when comparing chromium-treated cells at adjacent concentrations

To determine the cytotoxicity of chromium, we first determined the half maximal inhibitory concentration (IC_50_) of Cr(VI) in human neuronal (SK-N-SH) and human astrocyte (U-87 MG) cell lines using MTT assay and PrestoBlue assay across a concentration range of 1–1000 µM. Exposure to Cr(VI) at submicromolar concentrations resulted in a concentration-dependent decrease in cell viability in both SK-N-SH and U-87 MG cells (Fig. 1A and B). The viability of neurons was substantially more reduced than that of astrocytes, with the latter being more than twice as viable after incubation with 10 µM Cr(VI) for 24 h (viability: 17.98 ± 3.30% in SK-N-SH cells vs. 41.66 ± 6.44% in U-87 MG cells by MTT assay, and 2.00 ± 0.52% in SK-N-SH cells vs. 45.95 ± 5.33% in U-87 MG cells by PrestoBlue assay). We performed cell viability assay in primary human neurons and astrocytes to validate the cell line findings. Consistently, exposure to Cr(VI) resulted in a greater reduction in viability in primary human neurons than in primary human astrocytes (IC_50_ at 24 h: 24.55 µM vs. 32.05 µM), and a similar trend was observed in cell lines (2.50 µM in SK-N-SH cells vs. 10.79 µM in U-87 MG cells) (Fig. 1B). Furthermore, LDH cytotoxicity assay showed a significant increase in LDH release in primary human neurons at 10 µM Cr(VI), whereas primary human astrocyte required a higher concentration (100 µM) (Fig. 1C). The results showed that neurons are more sensitive to Cr(VI) than astrocytes; however, to further investigate the mechanisms underlying Cr(VI) effects, the concentrations used in this study were designed based on the IC_50_ values of astrocytes to better mimic conditions in which both cell types are exposed to Cr(VI) concurrently.

We next investigated Cr(VI) influx into neurons and astrocytes using flame atomic absorption spectroscopy. Concentration–and time–dependent increases in the intracellular accumulation of Cr(VI) were observed in both SK-N-SH and U-87 MG cells after Cr(VI) exposure (Fig. 1D). The results showed that more Cr(VI) deposited in neurons than in astrocytes at 24 h after exposure to 2.5 and 5 µM Cr(VI) (240.63 ± 38.42 and 472.41 ± 43.04 µg/g wet weight in SK-N-SH cells and 112.81 ± 31.81 and 351.34 ± 45.39 µg/g wet weight in U-87 MG cells, respectively).

### Cr(VI)-Induced Apoptotic Cell Death in Neurons and Astrocytes

**Fig. 2:**
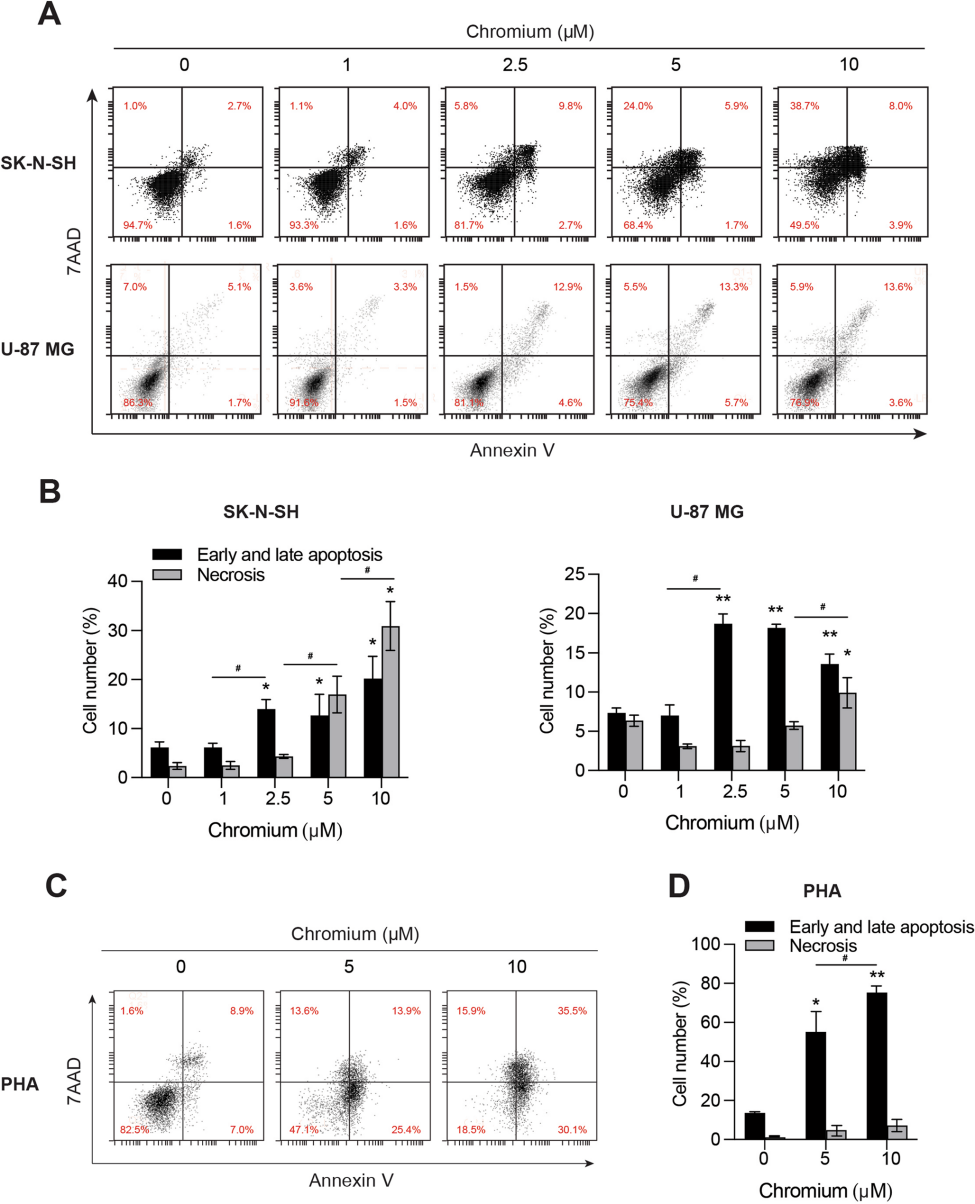
Cr(VI) induced apoptosis in human neuronal (SK-N-SH) and human astrocyte (U-87 MG) cell lines, as well as primary human astrocytes (PHA). **A–B** The levels of apoptotic and necrotic cells among SK-N-SH and U-87 MG cells were assessed by flow cytometry using Annexin V and 7–AAD staining after 24 h of exposure to Cr(VI) at concentrations of 1–10 µM (*n* = 4). Representative dot plots showing an increase of cells in the lower right quadrant (Annexin V^+^/7−AAD^−^ cells; early apoptotic cells), the upper right quadrant (Annexin V^+^/7−AAD^+^ cells; late apoptotic cells), and the upper left quadrant (Annexin V^−^/7−AAD^+^ cells; necrotic cells) in both cell types. **C–D** The levels of apoptotic and necrotic cells among PHA cells were assessed by flow cytometry using Annexin V and 7**–**AAD staining after 24 h of exposure to Cr(VI) at concentrations of 5 and 10 µM (*n* = 4). Representative dot plots and bar graph showing concentration**–**dependent increases in early and late apoptotic cells but not in necrotic cells. All data are presented as mean ± SEM. Statistically significant differences are shown as *p < 0.05 and **p < 0.01, compared with mock-treated cells and ^#^*p* < 0.05 when comparing chromium-treated cells at adjacent concentrations. 7**–**AAD, 7**–**aminoactinomycin D

To evaluate differences in the types of cell death induced by Cr(VI), we assessed the apoptotic pathway using flow cytometry with Annexin V and 7–AAD staining. After 24 h of exposure to 10 µM Cr(VI), the numbers of early and late apoptotic cells (Annexin V^+^) and necrotic cells (Annexin V^−^/7–AAD^+^ cells) increased by approximately 20% among both SK-N-SH and U-87 MG cells (Fig. 2A and B). In contrast Cr(VI), prominently triggered apoptotic cell death but not necrosis in PHA (Fig. 2C).

**Fig. 3:**
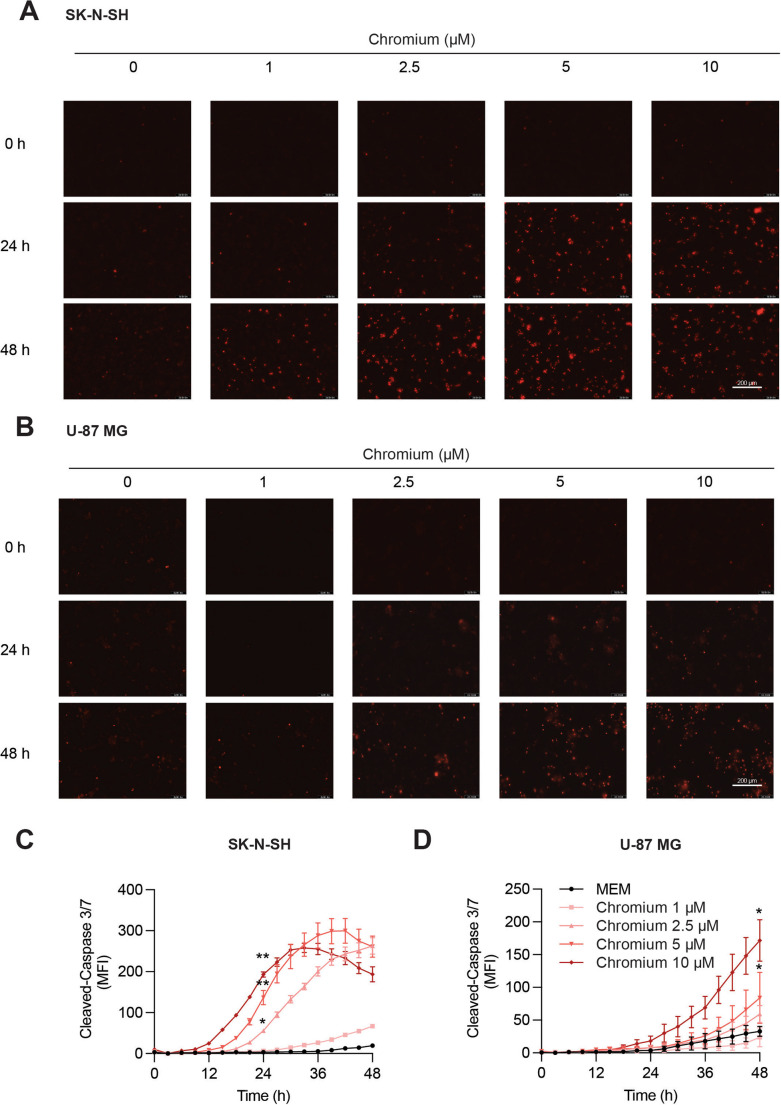
Cr(VI) **–**induced caspase-dependent apoptotic pathway in human neuronal (SK-N-SH) and human astrocyte (U-87 MG) cell lines. The levels of cleaved–caspase 3/7 in SK-N-SH and U-87 MG cells were determined by live cell imaging using IncuCyte^®^ Caspase 3/7 dye staining following exposure to Cr(VI) at concentrations of 1–10 µM for up to 48 h (*n* = 3). **A–B** Representative live cell images of caspase 3/7–labelled cells showing a time**–**and concentration**–**dependent increase in caspase 3/7 immunoreactivity (red) in both cell types. **C–D** Quantification of MFI corresponding to images in panels A and B. All data are presented as mean ± SEM. Statistically significant differences are shown as *p < 0.05 and **p < 0.01, compared with mock**–**treated cells at the same timepoint. The scale bar represents 200 μm. MFI, mean fluorescent intensity

**Fig. 4:**
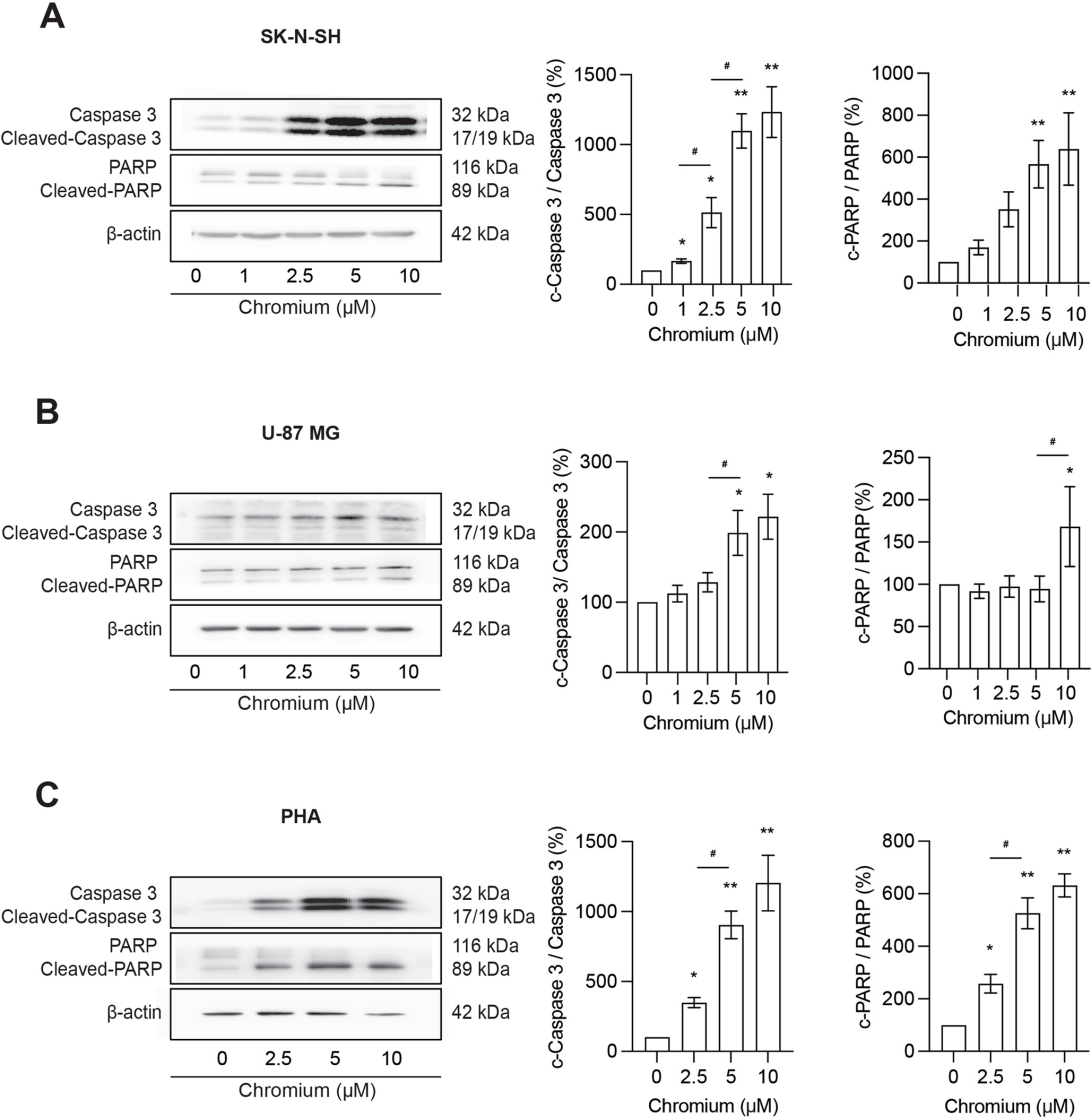
Cr(VI) induced the cleavage of caspase 3 and PARP in human neuronal (SK-N-SH) and human astrocyte (U-87 MG) cell lines, as well as primary human astrocytes (PHA). The levels of apoptotic markers, cleaved–caspase 3 and cleaved–PARP, were determined by western blotting in **A** SK-N-SH, **B** U-87 MG, and **C** PHA cells after 24 h of exposure to Cr(VI) (*n* = 5 for SK-N-SH and U-87 MG cells and *n* = 3 for PHA). SK-N-SH and U-87 MG cells were treated with 1–10 µM Cr(VI), while PHA cells were exposed to 2.5–10 µM. Cr(VI) increased the levels of cleaved–caspase 3 and cleaved–PARP in both cell types following Cr(VI) exposure at relevant concentrations. All data are presented as mean ± SEM. Statistically significant differences are shown as *p < 0.05 and **p < 0.01, compared with mock**–**treated cells and ^#^*p* < 0.05 when comparing chromium**–**treated cells at adjacent concentrations. PARP, poly(ADP**–**ribose) polymerase

We next determined the expression of caspase 3/7, a key component of the apoptotic pathway. Live cell imaging analysis detected time– and concentration–dependent increases in the number of caspase 3/7–labelled cells in both SK-N-SH and U-87 MG cells after exposure to Cr(VI) (Fig. 3A and C). In addition, an increase in the mean fluorescent intensity of cleaved–caspase 3/7 was initially observed at 24 h in SK-N-SH cells but at 48 h in U-87 MG cells. We further assessed the expression of cleaved–caspase 3 and cleaved–poly (ADP–ribose) polymerase 1 (PARP1), a well–established substrate of caspase 3. After 24 h of exposure to 2.5–10 µM Cr(VI), the expression levels of cleaved–caspase 3 and cleaved–PARP were elevated in all three cell types, SK-N-SH, U-87 MG and PHA cells, particularly at Cr(VI) concentrations of 5 and 10 µM (Fig. 4A and C).

### Cr(VI)-Induced Autophagic Cell Death in Neurons and Astrocytes

**Fig. 5:**
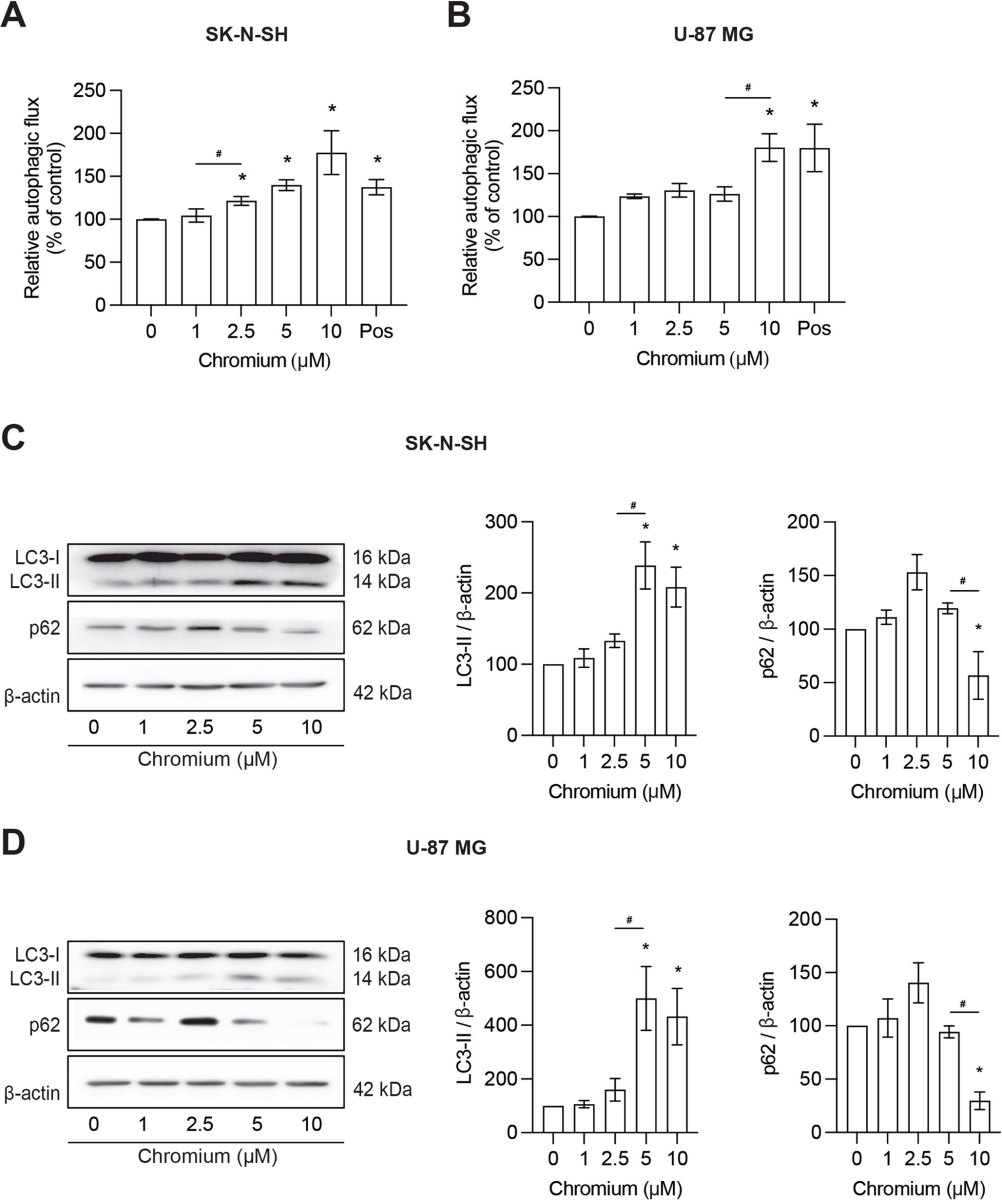
Cr(VI) induced autophagy in human neuronal (SK-N-SH) and human astrocyte (U-87 MG) cell lines. **A–B** The levels of autophagic flux in SK-N-SH and U-87 MG cells were detected by flow cytometry using CYTO**–**ID^®^ green staining following Cr(VI) exposure at 1–10 µM for 24 h (*n* = 4). In SK-N-SH cells, both low and high doses of Cr(VI) elevated autophagic flux, whereas only the high dose induced autophagic flux in U-87 MG cells. **C–D** The levels of autophagic markers, LC3 and p62, in SK-N-SH and U-87 MG cells were determined by western blotting after exposure to Cr(VI) at 1–10 µM for 24 h (*n* = 4). Cr(VI) induced autophagy by elevating LC3**–**II/LC3**–**I levels and decreasing p62 expression in both cell types. All data are presented as mean ± SEM. Statistically significant differences are shown as *p < 0.05 and **p < 0.01, compared with mock**–**treated cells and ^#^*p* < 0.05 when comparing chromium**–**treated cells at adjacent concentrations. LC3, light chain 3

To investigate the involvement of autophagy in Cr(VI)-induced neuronal cell death, we examined the formation of autophagic vesicles by labelling the cells with CYTO**–**ID^®^ red fluorescent dye. After 24 h of exposure to 2.5–10 µM Cr(VI), an increase in autophagic flux was observed in SK-N-SH cells, whereas U-87 MG cells exhibited this response only at 10 µM (Fig. 5A and B). We further assessed the expression of autophagy**–**related proteins, specifically light chain (LC)3 and LC3**–**binding protein p62 (a polyubiquitin**–**binding protein that facilitates LC3 degradation), in SK-N-SH and U-87 MG cells. A significant increase in LC3**–**II–a cleaved form of LC3–expression was observed, accompanied by a decrease in p62 levels, in both SK-N-SH and U-87 MG cells (Fig. 5C and D).

### Cr(VI)-Induced Brain Cell Death was Mediated by the MAPK Signalling Pathway

**Fig. 6:**
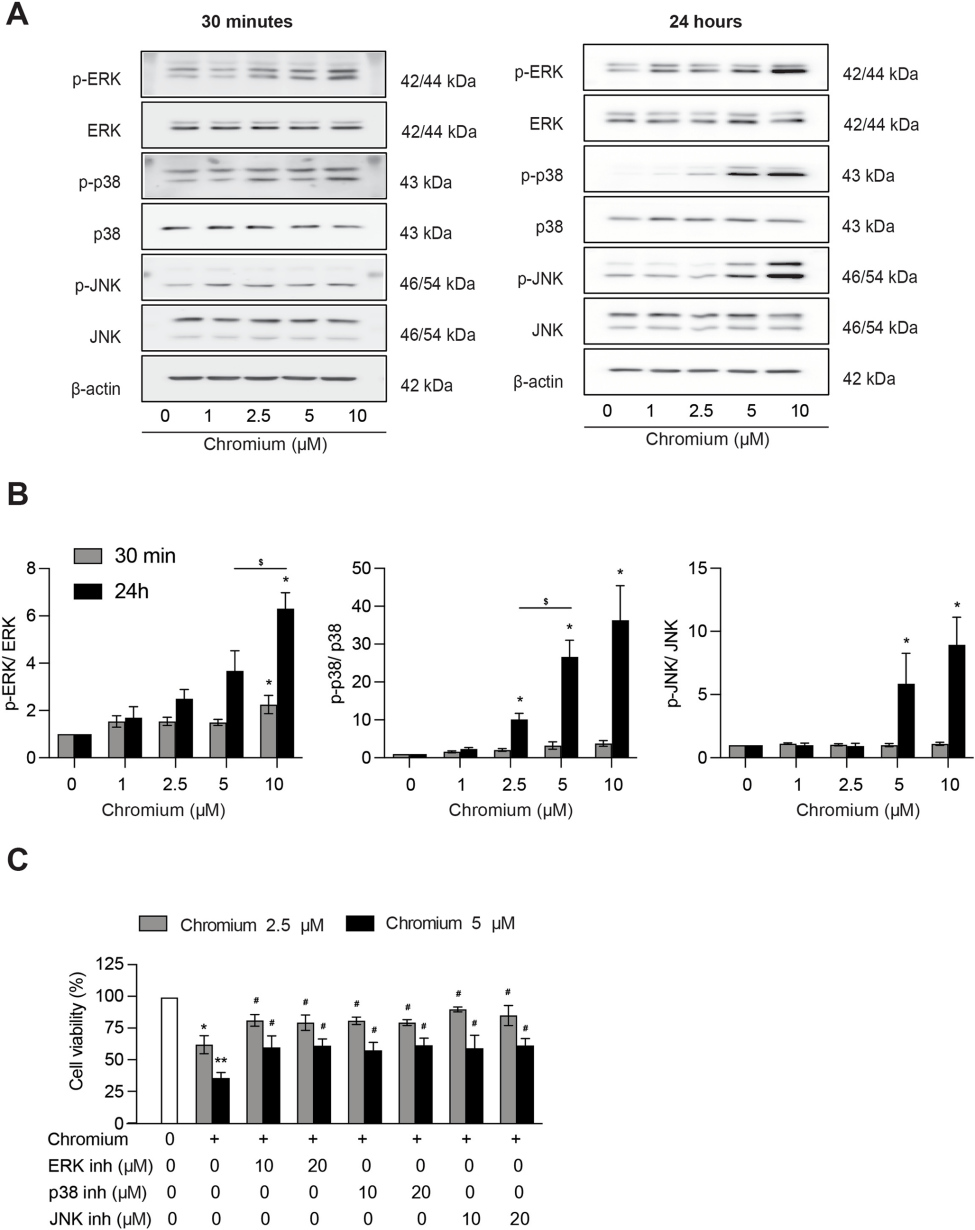
Cr(VI) induced cell death via MAPK pathway in human neuronal (SK-N-SH) cell line. **A–B** Expression levels of MAPK pathway components in SK-N-SH cells were determined by western blotting after exposure to Cr(VI) at 1–10 µM for 30 min and 24 h (*n* = 4). Representative blot images of MAPK protein expression and quantification of ratio of phospho/total MAPKs corresponding to images in panel (A) showing an increase in the phosphorylation levels of ERK, JNK, and p38 at 24 h, as well as in the phosphorylation levels of ERK at 30 min. **C** Viability of SK-N-SH cells was assessed using MTT assays after 24 h of co–treatment with Cr(VI) (2.5 and 5 µM) and selective MAPK inhibitors (10 and 20 µM) (*n* = 4). Inhibition of the MAPK pathway suppressed Cr(VI) –induced cell death in SK-N-SH cells. All data are presented as mean ± SEM. Statistically significant differences are shown as *p < 0.05 and **p < 0.01, compared with mock–treated cells, ^#^*p* < 0.05, compared between chromium–treated cells with and without specific MAPK inhibitors at the relevant concentration of chromium, and ^$^*p* < 0.05 when comparing chromium-treated cells at adjacent concentrations

**Fig. 7:**
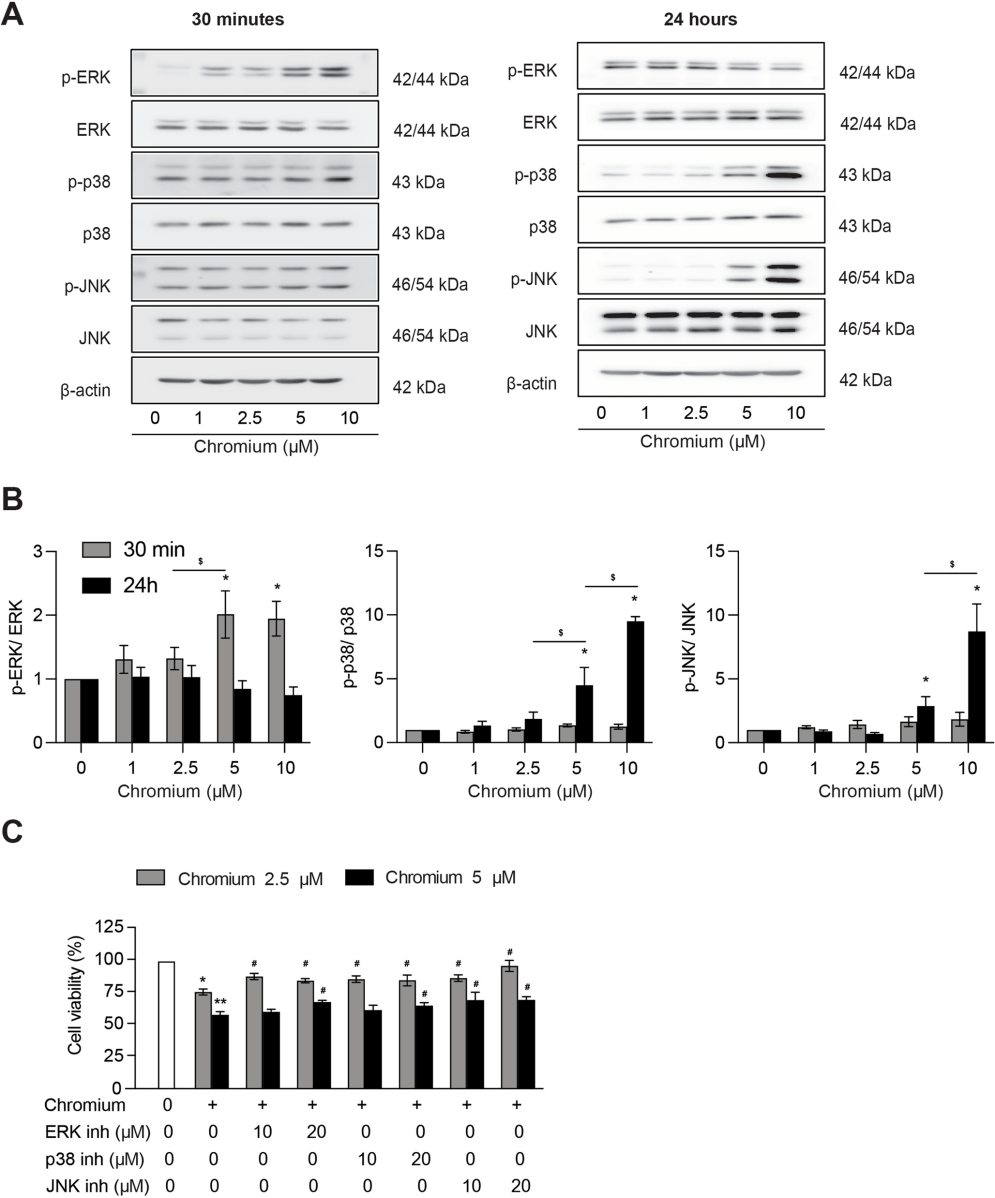
Cr(VI) induced cell death via MAPK pathway in human astrocyte (U-87 MG) cell line. **A–B** Expression levels of MAPK pathway components in U-87 MG cells were determined by western blotting after exposure to Cr(VI) at 1–10 µM for 30 min and 24 h (*n* = 4). Representative blot images of MAPK protein expression and quantification of ratio of phospho/total MAPKs corresponding to images in panel A showing an increase in the phosphorylation levels of ERK at 30 min and the phosphorylation levels of JNK and p38 at 24 h. **C** Viability of U-87 MG cells was assessed using MTT assays after 24 h of co–treatment with Cr(VI) (2.5 and 5 µM) and selective MAPK inhibitors (10 and 20 µM) (*n* = 4). Inhibition of the MAPK pathway suppressed chromium-induced cell death in U-87 MG cells. All data are presented as mean ± SEM. Statistically significant differences are shown as *p < 0.05 and **p < 0.01, compared with mock-treated cells, ^#^*p* < 0.05, compared between chromium–treated cells with and without specific MAPK inhibitors at the relevant concentration of chromium, and ^$^*p* < 0.05 when comparing chromium–treated cells at adjacent concentrations

We next investigated the signalling pathway involved in Cr(VI)**–**induced brain cell death. In SK-N-SH cells, Cr(VI) exposure increased the phosphorylation levels of ERK, JNK, and p38 at 24 h, as well as the phosphorylation level of ERK at 30 min (Fig. 6A and B), whereas in U-87 MG cells, phosphorylation of ERK was elevated at 30 min and phosphorylation of JNK and p38 was increased at 24 h (Fig. 7A and B). To verify that Cr(VI) indeed induces neuronal cell death by promoting the MAPK signalling pathway, specific MAPK inhibitors, namely, U0126 (MEK1/2 inhibitor), SP600125 (JNK inhibitor), and SB203580 (p38 inhibitor), were co**–**administered with Cr(VI). We observed that inhibition of the MAPK pathway suppressed Cr(VI) **–**induced brain cell death in both SK-N-SH and U-87 MG cells, suggesting that MAPK signalling plays a critical role in mediating Cr(VI) **–**induced neurotoxicity (Figs. 6C and 7C).

### Cr(VI) Arrested the Astrocyte Cell Cycle at the S Phase

**Fig. 8:**
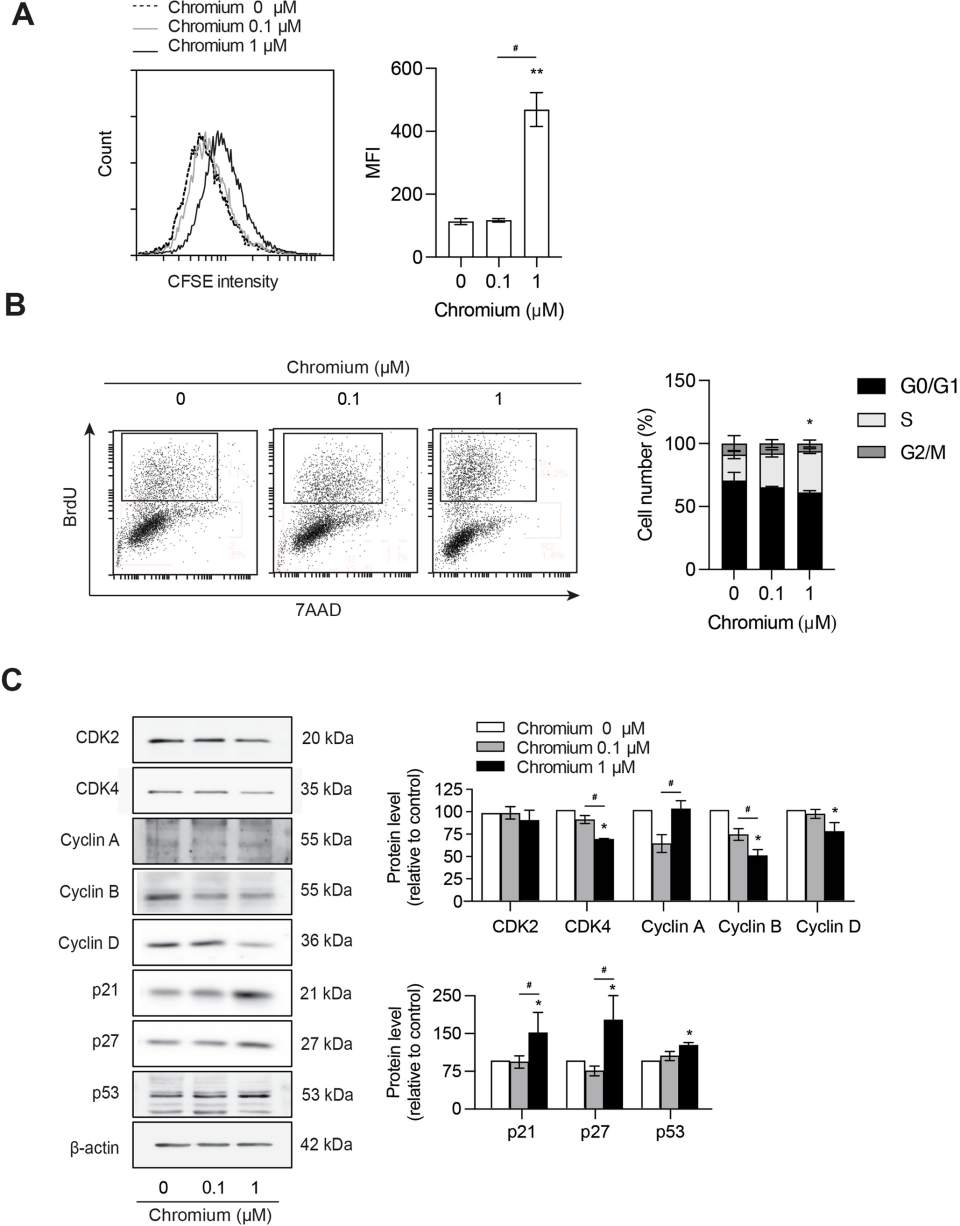
Cr(VI) induced cell cycle arrest in human astrocyte (U-87 MG) cell line. **A** Antiproliferative effects of Cr(VI) in U-87 MG cells were assessed by flow cytometry using CFSE staining (*n* = 3). A representative histogram showing the rightward shift in MFI of CFSE in U-87 MG cells after exposure to 1 µM Cr(VI) for 48 h. Dashed line, gray line, and black line represent untreated cells and cells treated with 0.1 and 1 µM Cr(VI), respectively. **B** The proportion of cells in each phase of the cell cycle was assessed by flow cytometry using BrdU staining (*n* = 3). Representative dot plots and a bar graph showing the increased proportion of cells in S phase among U-87 MG cells, accompanied by a decreased proportion of cells in G_0_/G_1_ and G_2_M phase after exposure to 1 µM Cr(VI) for 48 h. **C** Expression levels of cell cycle–related proteins in U-87 MG cells were determined by western blotting after exposure to chromium at 0.1 and 1 µM for 48 h (*n* = 4). Cr(VI) altered the expression levels of cell cycle regulators by increasing the levels of cell cycle inhibitors (p21, p27, and p53), accompanied by decreased levels of CDK4, cyclin B, and cyclin D. All data are presented as mean ± SEM. Statistically significant differences are shown as *p < 0.05 and **p < 0.01, compared with mock-treated cells and ^#^*p* < 0.05 when comparing chromium-treated cells at adjacent concentrations. CFSE, carboxyfluorescein succinimidyl ester; MFI, mean fluorescent intensity

The effects of Cr(VI) of inhibiting astrocyte proliferation were assessed using U-87 MG cells. Exposure to 1 µM Cr(VI) for 48 h arrested cell cycle progression and resulted in a significant increase in the proportion of cells in S phase from 20% in untreated cells to 35% in treated ones, as revealed by carboxyfluorescein diacetate succinimidyl ester (CFSE) analysis and 5–bromo–2’–deoxyuridine (BrdU) labelling (Fig. 8A and B). We further assessed the expression of cell cycle–regulating proteins critical for S-phase transition and progression. The results showed that the expression of CDK inhibitors, including p21, p27, and p53 were upregulated upon exposure to Cr(VI), whereas that of cyclin B, cyclin D, and CDK4 were downregulated, supporting the above finding that Cr(VI) governs the S phase cell–cycle progression (Fig. 8C).

## Discussion

The main finding of this study was that exposure to Cr(VI) exerted greater toxicity in human neurons than in astrocytes. The exposure of these cell types to Cr(VI) significantly reduced their viability in a concentration–dependent manner, with an IC_50_ of approximately 1 µM in neurons and 5 µM in astrocytes at 24 h after exposure. The cytotoxic effects of Cr(VI) on neurons were mediated by a pathway similar to that of astrocytes. Specifically, Cr(VI) exposure induced a DNA damage response that led to apoptosis as well as autophagy with cell cycle arrest at the S phase. Cr(VI) exposure also activated MAPK signalling pathways, which was reinforced by the finding that the inhibition of these pathways using specific inhibitors resulted in a substantial increase in cell viability. In contrast, chromium–mediated DNA damage did not result from reactive oxygen species (ROS)–driven mechanisms (Supplementary Fig. S1).

In this study, neurons were found to be more susceptible to Cr(VI) toxicity than astrocytes. This same pattern has been reported for other toxic substances such as copper [20] and unconjugated bilirubin [21]. Cr(VI) has also been shown to exert toxic effects on human lung cells at concentrations included among those analysed in the present study (1–10 µM) [22]. Despite much that remains unknown about Cr(VI) transport into brain cells, it is conceivable that Cr(VI) enters cells by facilitated diffusion through nonspecific anion channels (mostly phosphate or sulphate channels) [7]. In addition, our results demonstrated that the average intracellular uptake level of Cr(VI) in neurons and astrocytes after 24 h of exposure to 10 µM Cr(VI) was approximately 450 µg/g wet weight. In autopsied brain tissues of healthy human subjects, average concentrations of chromium ranged from 4.7 to 136 ng/g wet weight, indicating that the concentrations used in our study exceed physiological levels and could lead to adverse effects on human health [23]. In addition, the concentrations shown in our study to impair neuronal and astrocyte viability are relevant to the average concentrations of Cr(VI) reported in brain tissues of individuals residing in polluted areas of Mexico City (910 ± 63 µg/g dry tissue) [24].

Cr(VI)–induced alterations in the expression of proteins involved in DNA damage, mitochondria–mediated apoptosis, and autophagy in human neurons and astrocytes. DNA damage–induced autophagy has been shown to be associated with both pro–survival and cell death–promoting pathways [25]. Specifically, autophagy can act as a protective mechanism against DNA damage, promoting cellular survival by removing damaged components. However, excessive autophagy can lead to apoptosis, preventing the propagation of damaged cells [26]. Accumulating evidence indicates that apoptosis and autophagy act as dual mechanisms contributing to neuronal impairments [27]. In line with this, Cr(VI) has been reported to induce kidney damage in rats by promoting apoptosis and autophagy via mitochondrial dysfunction and disrupted mitochondrial dynamics [28]. In addition, PARP1–activated autophagy has been shown to impair the function of human keratinocytes and human renal cells in response to Cr(VI) [29, 30]. Similarly, our results showed that the exposure of human neurons and astrocytes to Cr(VI) prominently increased the expression of PARP, likely triggered by an increase in cleaved–caspase 3/7. This in turn resulted in the induction of autophagy, as evidenced by an increase in LC3–II/LC3–I levels and a decrease in p62 levels. Nevertheless, the precise mechanisms by which autophagy acts as a pro–survival or pro–death mechanism require further in–depth investigation. To confirm this, treatments with specific autophagy inhibitors should be employed in future experiments. In addition, animal studies may further strengthen our conclusions.

The caspase 3/MAPK signalling pathway plays a critical role in the downstream regulation of the death of brain cells induced by Cr(VI). Excessive activation of the MAPK pathway stimulates cytochrome c release and caspase 3/7 activation [31]. MAPK activation is also associated with toxin–induced neurodegenerative diseases and has been proposed as a crucial therapeutic target for chronic inflammatory diseases and neurodegenerative diseases [32, 33]. Cr(VI) –induced apoptotic cell death has been shown to occur via the activation of JNK and p38, but not ERK, in murine embryonic stem cells [34] and non–small–cell lung carcinoma cells [35], whereas in primary rat neurons, all three MAPKs, namely JNK, p38, and ERK, are implicated [16]. Moreover, a recent study in human neuroblastoma SH-SY-5Y cells reported that Cr(VI)–induced ROS result in caspase 3–dependent apoptosis through the ERK1/2/AKT/AMPK pathway [15]. Consistent with previous studies, exposure to Cr(VI) in the present study significantly increased the phosphorylation of all MAPKs and elevated the levels of cleaved– caspase 3 in neurons and astrocytes. Moreover, the use of specific MAPK inhibitors markedly reduced Cr(VI) –induced cell death, as demonstrated by MTT assays. Thus, it has been hypothesised that Cr(VI) induces neuronal toxicity by activating MAPK/caspase 3 pathways.

In the present study, Cr(VI) enhanced G_1_/S phase transition and arrest of the cell cycle in S phase in human astrocytes. Compare with other heavy metals, such as cadmium, Cr(VI) elicited anti–proliferative effects in human astrocytes at lower concentrations than those required for cadmium (1 µM vs. 10 µM) [36]. Previous studies have shown that cell cycle arrest caused by Cr(VI) depends on the specific cell type and the level of exposure. For example, a low dose of Cr(VI) (4 µM) affected the G_1_/S transition in human hepatocytes, whereas higher doses (16–32 µM) led to G_2_/M phase arrest [37]. In contrast, a comparative study of human skin and lung cells demonstrated that Cr(VI) induced G_1_ phase arrest in skin cells and G_2_/M phase arrest in lung cells [38]. Moreover, the anti–proliferative effects of Cr(VI) during S phase have been reported to be associated with the increased expression of DNA damage–associated proteins, including ataxia telangiectasia mutated (ATM), gamma–H2A.X variant histone (H2AX), and proliferating cell nuclear antigen (PCNA) [22, 39, 40], resulting in DNA damage. Consistent with these previous findings, Cr(VI) induced DNA damage in our study, resulting in increased expression of CDK inhibitors, including p21, p27, and p53. This inhibition resulted in reductions in the levels of cell cycle–regulating proteins, including cyclin B, cyclin D, and CDK4. These proteins normally promote the activity of the cyclin A/CDK2 complex; thus, their reduction negatively affects this complex, delaying the S to G_2_/M transition and leading to the accumulation of cells in the S phase. Taking these findings together, the induction of cell cycle arrest by Cr(VI) may be directly caused by DNA damage and/or indirectly caused by the alteration in the expression levels of cell cycle–related proteins.

Cr(VI) is recognized as a highly toxic metal pollutant frequently detected in fine particulate (PM_2.5_). Exposure to PM_2.5_–bound Cr(VI) pollution potentially contributes to carcinogenic health risks [41] and impairment of human airway epithelial cells [42], human embryonic kidney cells [43], and human hepatocellular carcinoma cells [43]. However, no studies have specifically reported the impacts of PM_2.5_–bound Cr(VI) on brain cells, and only a few have examined the general effects of PM_2.5_. Learning and memory impairments linked to hippocampal dysfunction in rats exposed to PM_2.5_ [44], along with neurotoxicity observed in human SH-SY-5Y neuronal cells following PM_2.5_ exposure [45], suggest potential mechanisms by which Cr(VI) may contribute to PM_2.5_–induced neurotoxicity. However, elucidating the precise mechanisms by which PM_2.5_–bound Cr(VI) induces brain cell death remains challenging due to the complex and variable composition of PM_2.5_.

Although the present study elicits many insights, it also has some limitations. One limitation is the use of SK-N-SH and U-87 MG cell lines as models of neuron–and astrocyte–like cells. These cell lines are widely used to establish in vitro neurotoxicology models as alternatives to primary cells, since obtaining primary human neurons and astrocytes from healthy donors is limited and remains a significant challenge [46]. However, we also included primary human neurons and primary human astrocytes in cytotoxicity studies to partially address Cr(VI) toxicity.

## Conclusions

In this study, neurons were more susceptible than astrocytes to toxicity induced by Cr(VI). Cr(VI) caused neurotoxicity through the induction of DNA damage, mitochondria–mediated and caspase-dependent apoptosis, autophagy, and S–phase cell cycle arrest via MAPK activation. These results imply that cell signalling, cell proliferation, and apoptosis are disrupted by Cr(VI) poisoning, which may serve as an occupational and environmental risk factor for the development of neurological disorders.

## Supplementary Information

Below is the link to the electronic supplementary material.

Supplementary Material 1

Supplementary Material 2

## Data Availability

Data will be made available on request.
