# Supplementary material for: Hexavalent Chromium Induces Apoptosis and Autophagy in Human Neurons and Astrocytes via MAPK Pathway Activation
Source: Biol Trace Elem Res. 2026 Apr 5;204(7):5481–98. doi: 10.1007/s12011-026-05046-0 (PMC13319247; doi:10.1007/s12011-026-05046-0)
Supplement: Supplementary file 1 — Supplementary Material 1 [file 12011_2026_5046_MOESM1_ESM.docx]

**Hexavalent Chromium Induces Apoptosis and Autophagy in Human Neurons and Astrocytes via MAPK Pathway Activation**

Suttinee Phuagkhaopong^1^, Ratchanon Sukprasert^2^, Rapeewan Settacomkul^2^, Dusadee Ospondpant^3^, Kran Suknuntha^2,4^, Phisit Khemawoot^2,4^, Christopher Power^3^, Pornpun Vivithanaporn^2,4^

^1^Department of Pharmacology, Faculty of Medicine, Chulalongkorn University, Bangkok, Thailand

^2^Chakri Naruebodindra Medical Institute, Faculty of Medicine Ramathibodi Hospital, Mahidol University, Samut Prakan, Thailand

^3^Department of Medicine, University of Alberta, Edmonton, Alberta, Canada

^4^Ramathibodi Medical School, Faculty of Medicine Ramathibodi Hospital, Mahidol University, Samut Prakan, Thailand

Corresponding author

Pornpun Vivithanaporn

Chakri Naruebodindra Medical Institute, Faculty of Medicine Ramathibodi Hospital, Mahidol University, Samut Prakan, Thailand 10540 *E-mail address:* [pornpun.viv@mahdiol.ac.th](mailto:pornpun.viv@mahdiol.ac.th) (P. Vivithanaporn).

**
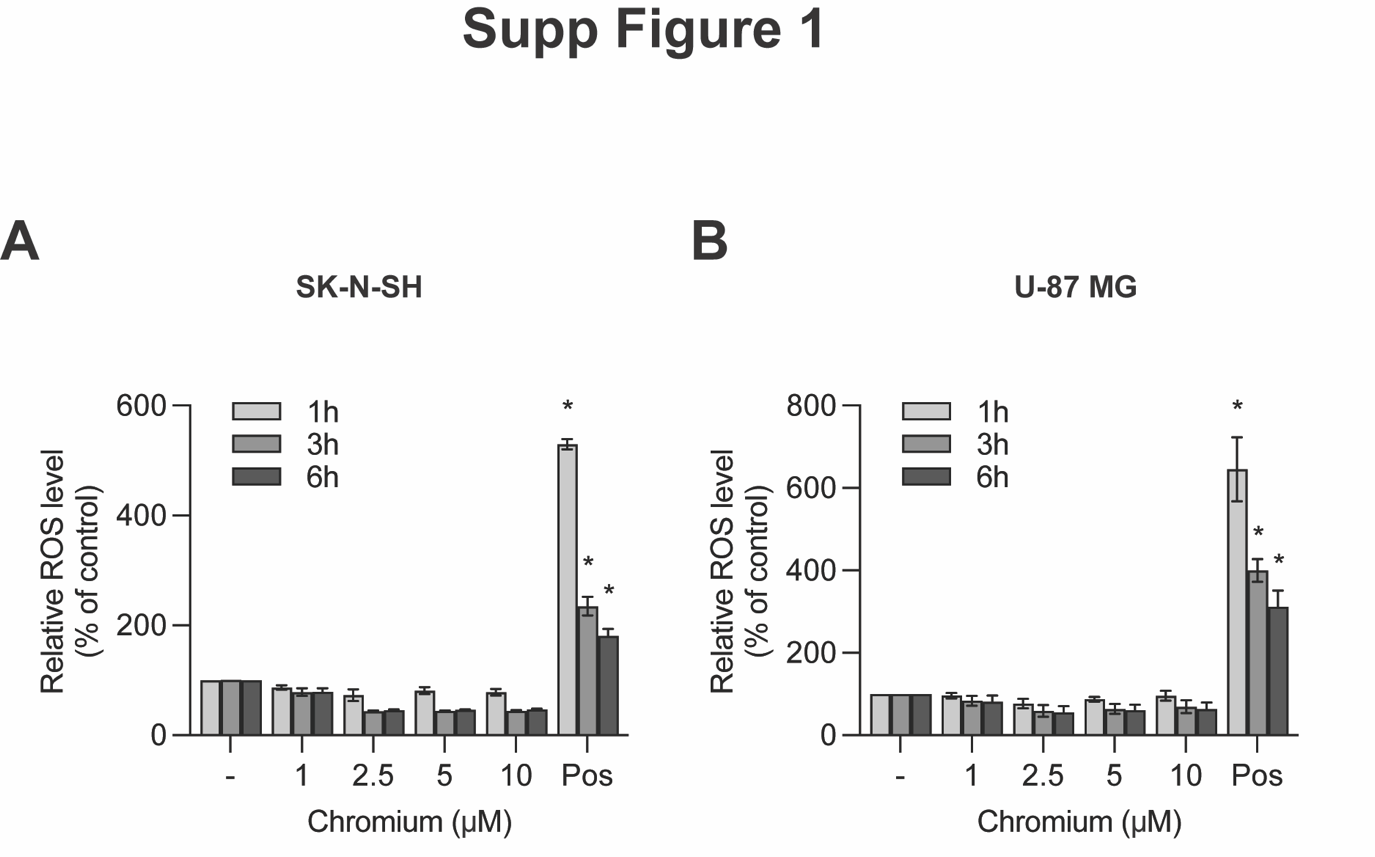
**

**Supplementary Fig. S1.** Chromium–induced ROS-independent cell death pathway in human neuronal (SK-N-SH) and human astrocyte cell lines (U-87 MG). **A, B** The levels of ROS in SK-N-SH and U-87 MG cells were determined by DCF fluorescence following exposure to chromium at 1–10 μM for 1, 3, and 6 h. Chromium did not induce ROS production in either cell type, even at high concentrations. Hydrogen peroxide (H_2_O_2_) was used as a positive control. All data are presented as mean ± SEM from three to five independent experiments. Statistically significant differences are shown as *p < 0.05, compared with mock–treated cells at the same timepoint. DCF, dichlorofluorescein; ROS, reactive oxygen species; Pos, positive control.
