# Supplementary material for: Hexavalent Chromium Induces Apoptosis and Autophagy in Human Neurons and Astrocytes via MAPK Pathway Activation
Source: Biol Trace Elem Res. 2026 Apr 5;204(7):5481–98. doi: 10.1007/s12011-026-05046-0 (PMC13319247; doi:10.1007/s12011-026-05046-0)

Images of Western Blotting for Figure 4A

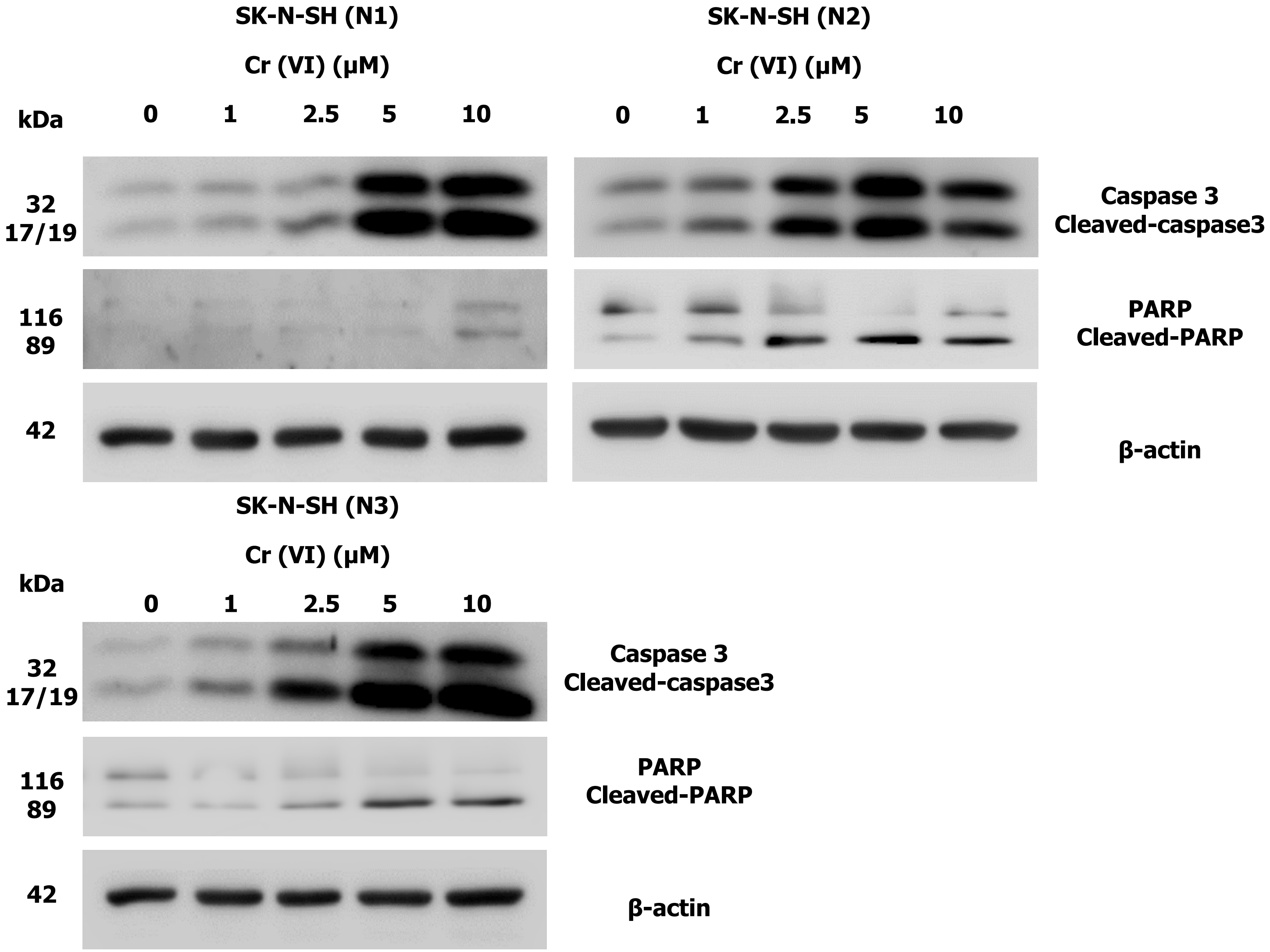

Images of Western Blotting for Figure 4B

U-87 MG (N1)

Cr (VI) (μM)

kDa 0 1 2.5 5 10

32
17/19
116
89
42

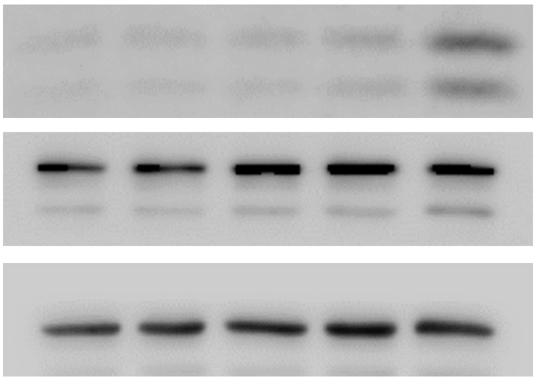

U-87 MG (N2)

Cr (VI) (μM)

0 1 2.5 5 10

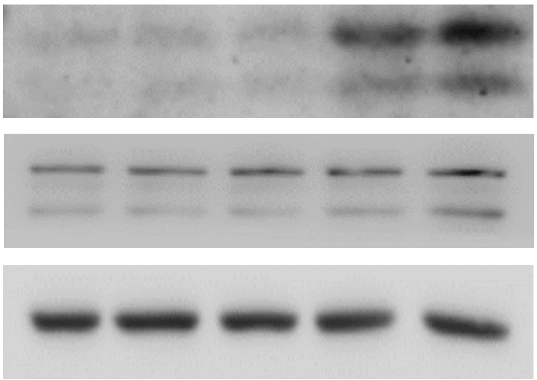

Caspase 3
Cleaved-caspase3

PARP
Cleaved-PARP

β-actin

U-87 MG (N3)

Cr (VI) (μM)

kDa 0 1 2.5 5 10

32
17/19
116
89
42

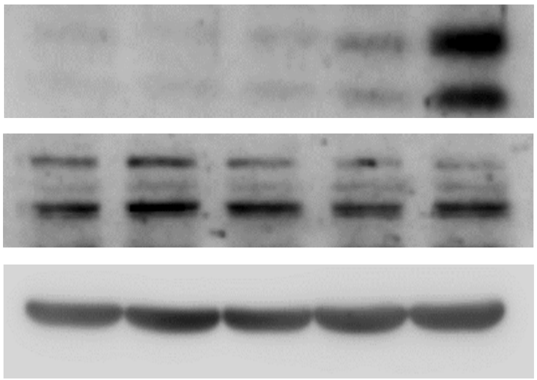

Caspase 3
Cleaved-caspase3

PARP
Cleaved-PARP

β-actin

Images of Western Blotting for Figure 4C

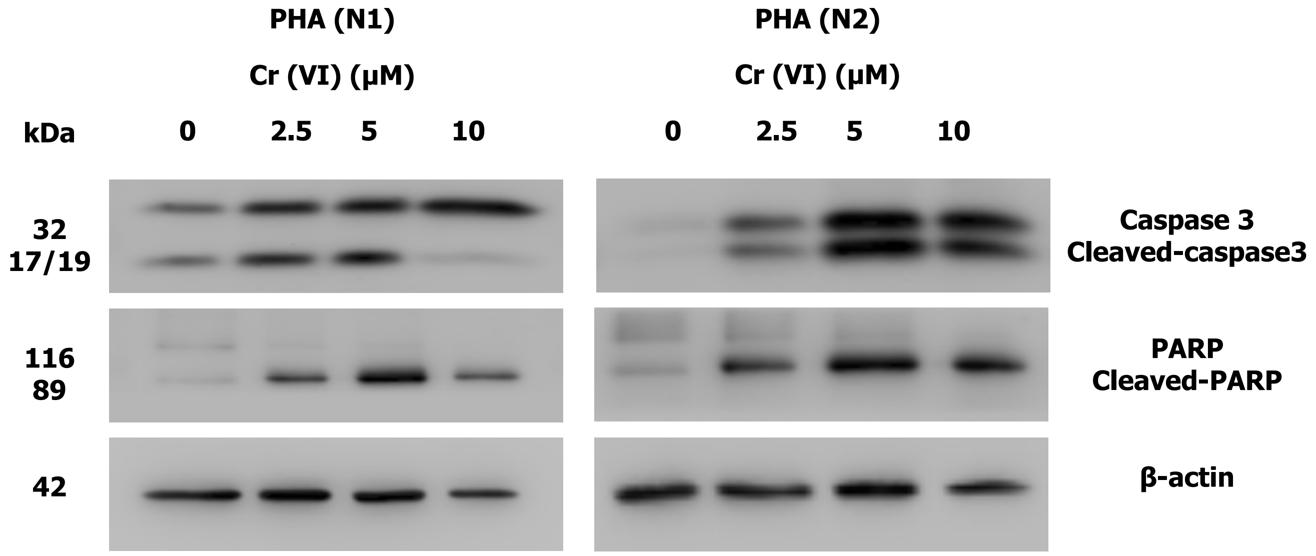

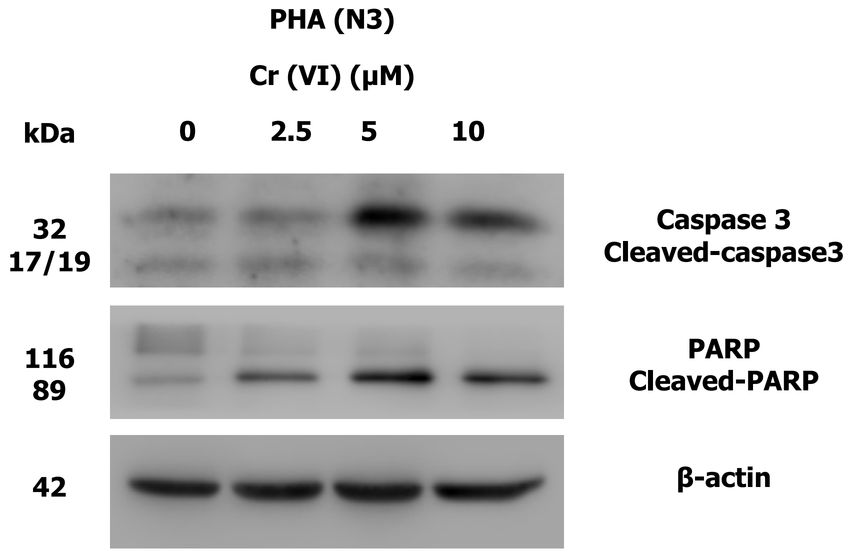

Images of Western Blotting for Figure 5C

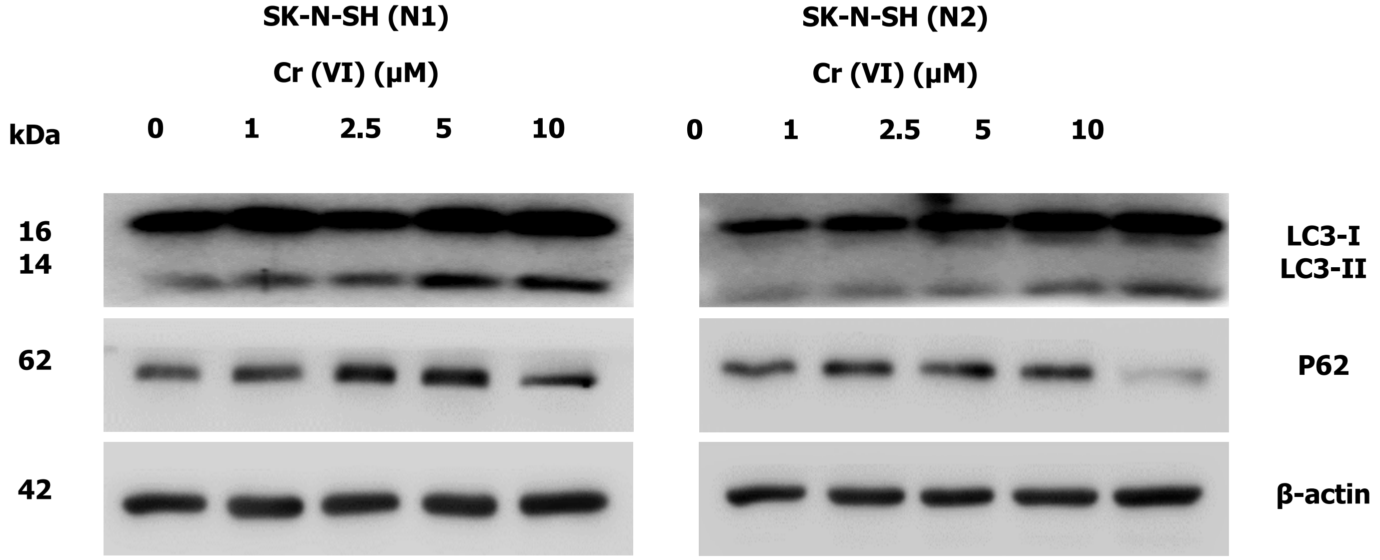

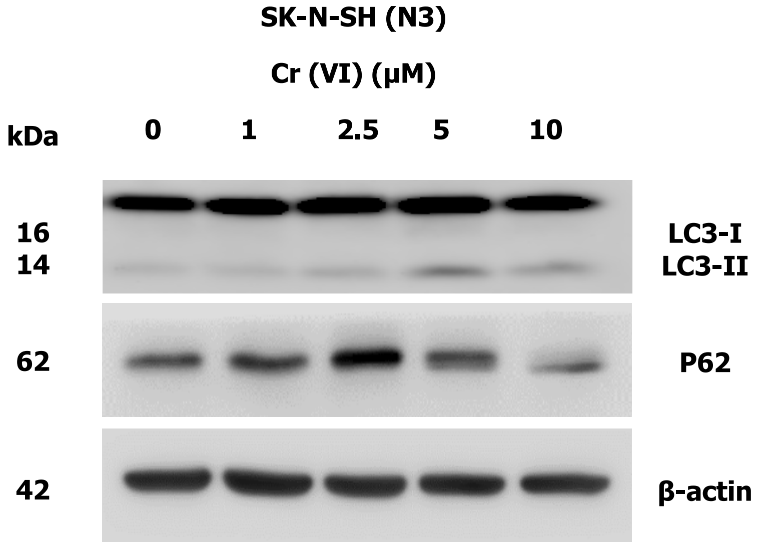

Images of Western Blotting for Figure 5D

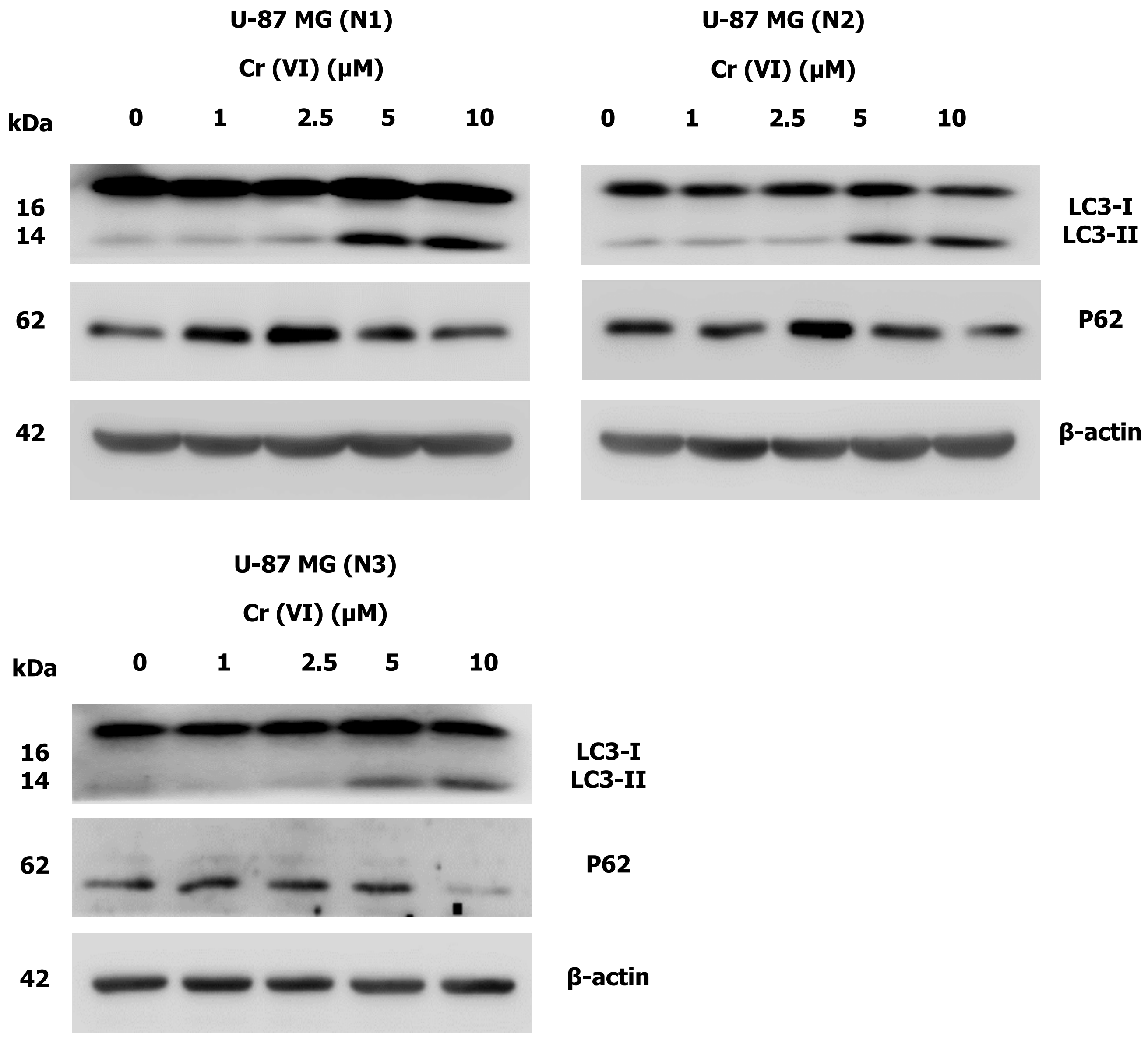

Images of Western Blotting for Figure 6A

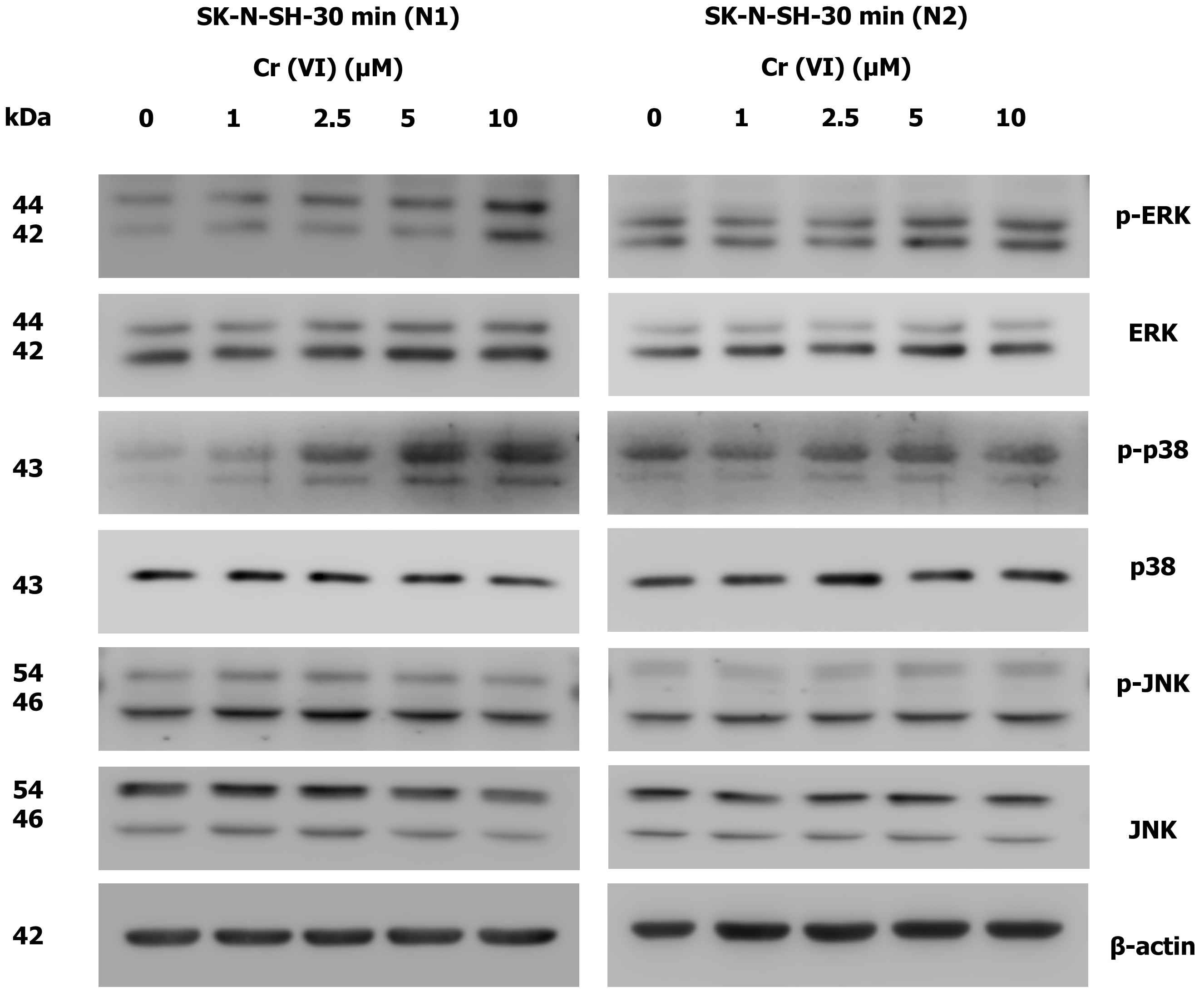

Images of Western Blotting for Figure 6A

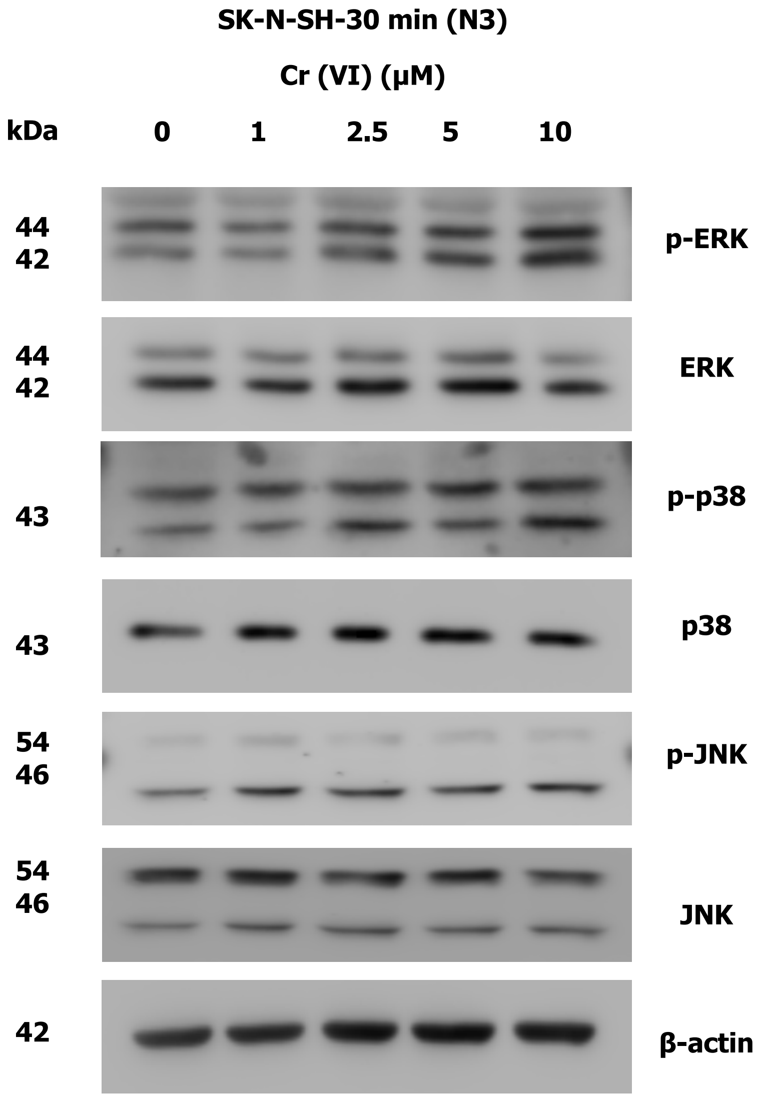

Images of Western Blotting for Figure 6A

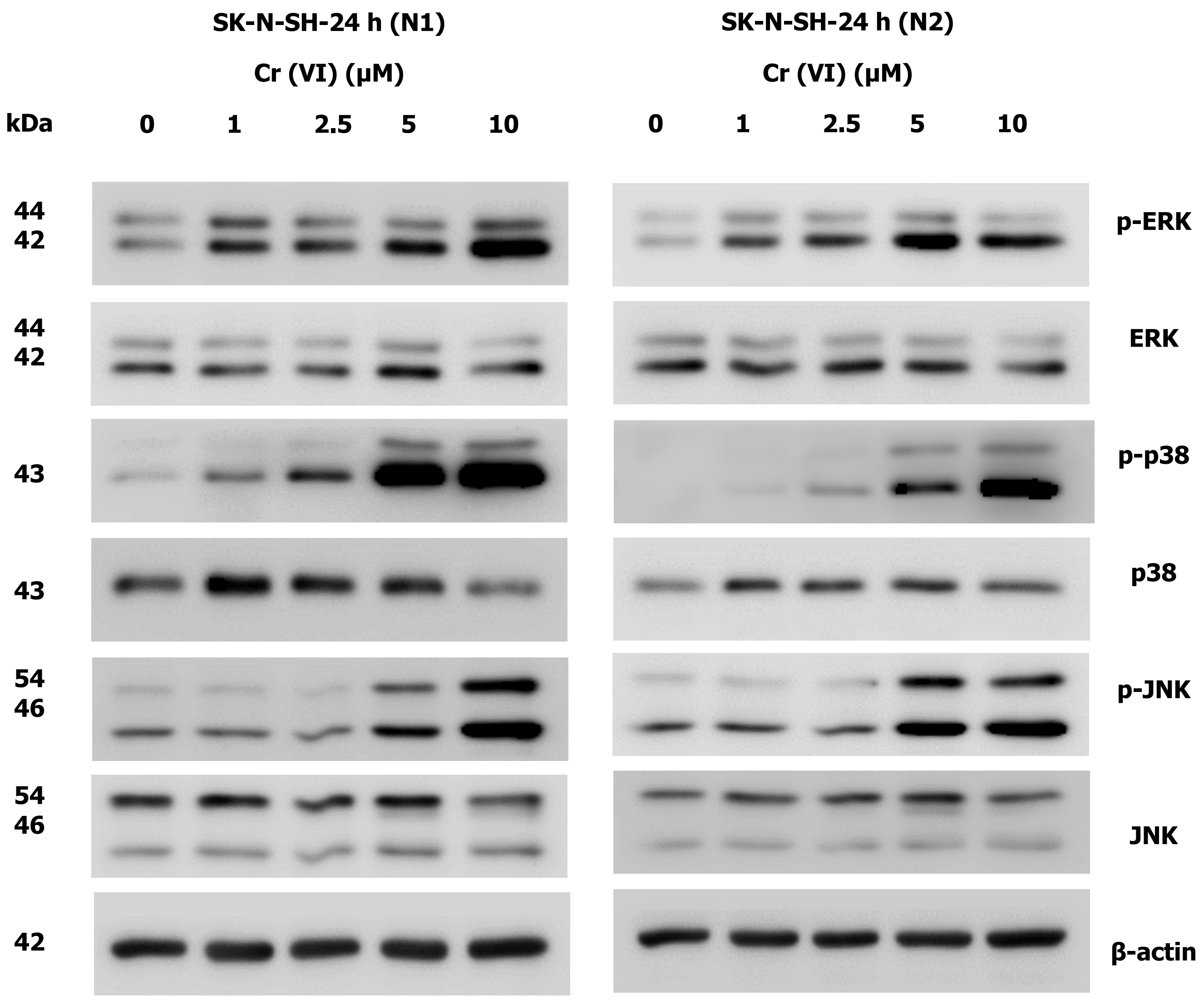

Images of Western Blotting for Figure 6A

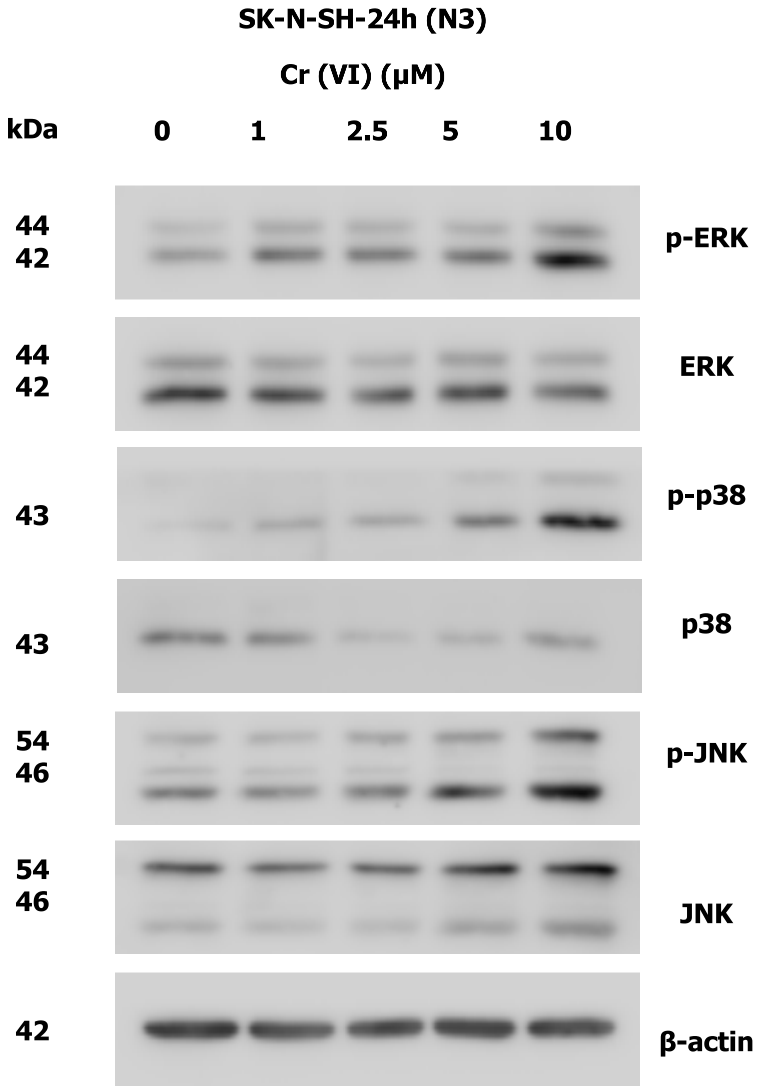

Images of Western Blotting for Figure 7A

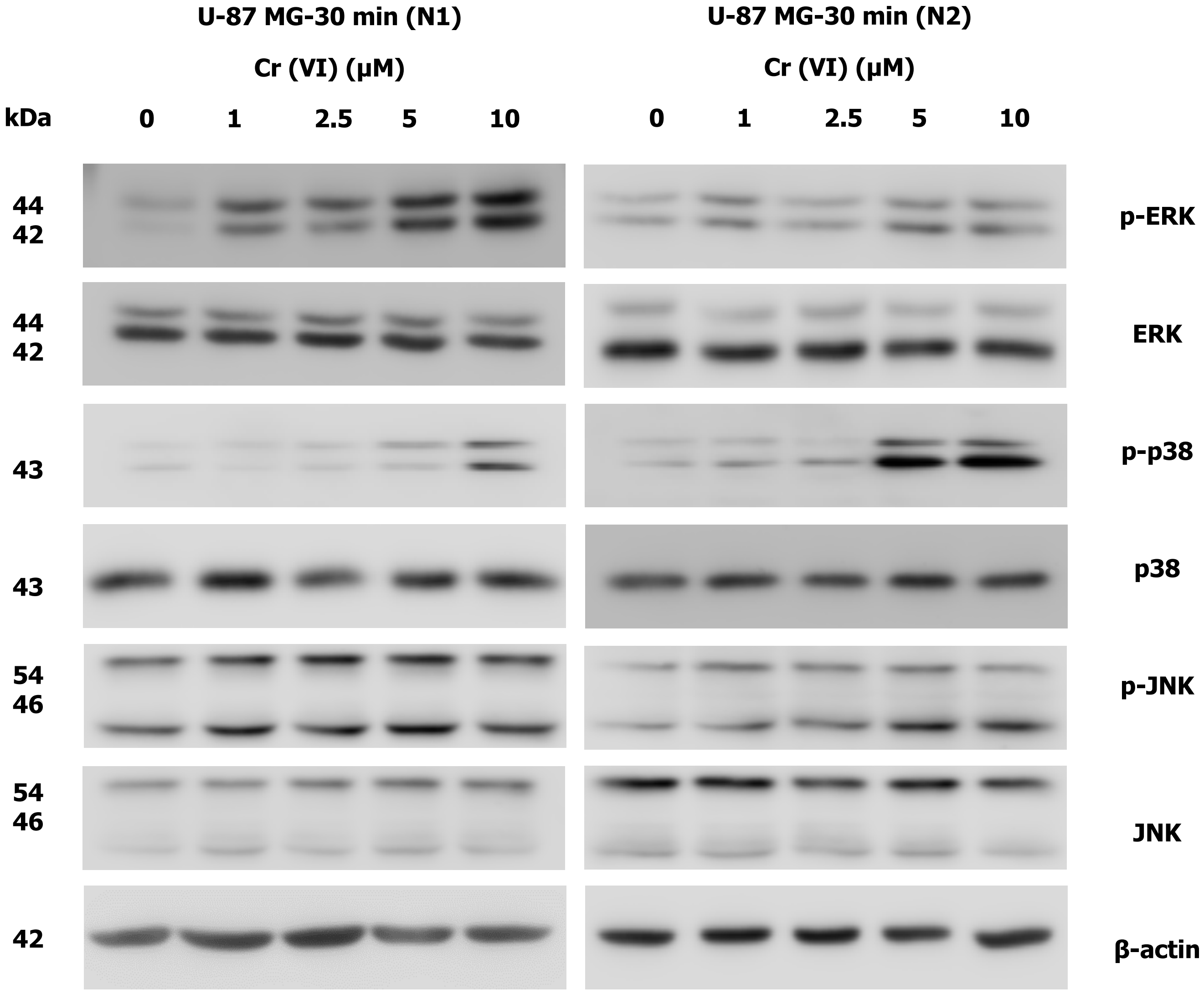

Images of Western Blotting for Figure 7A

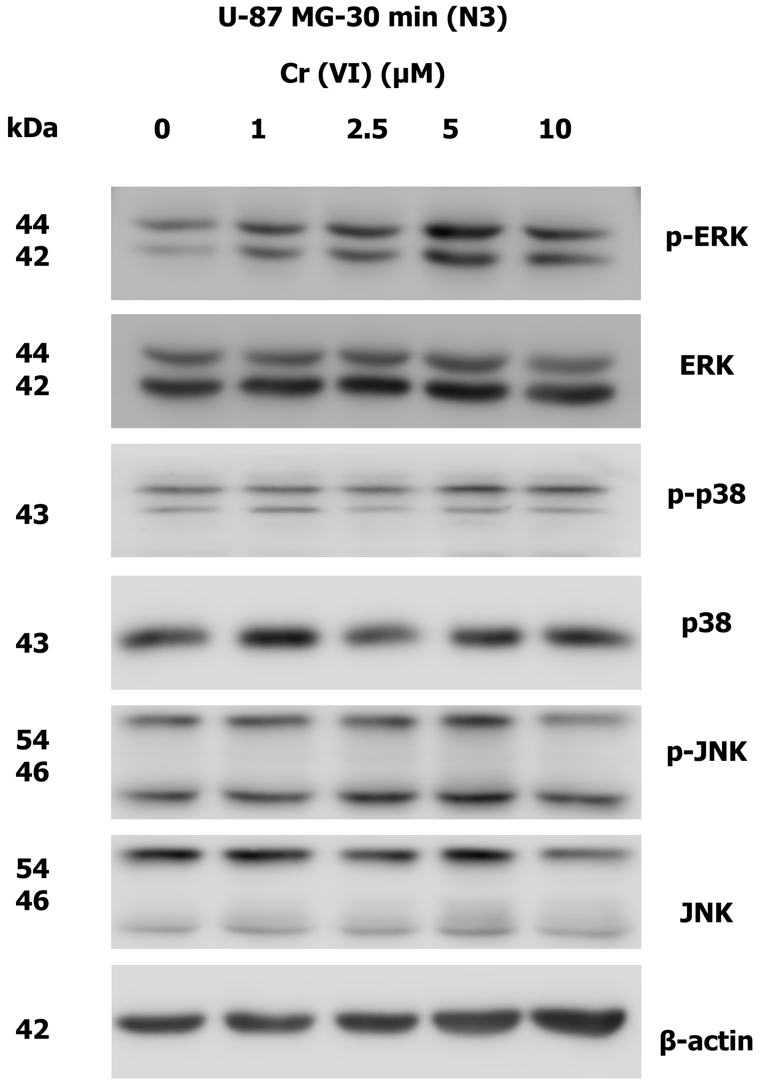

Images of Western Blotting for Figure 7A

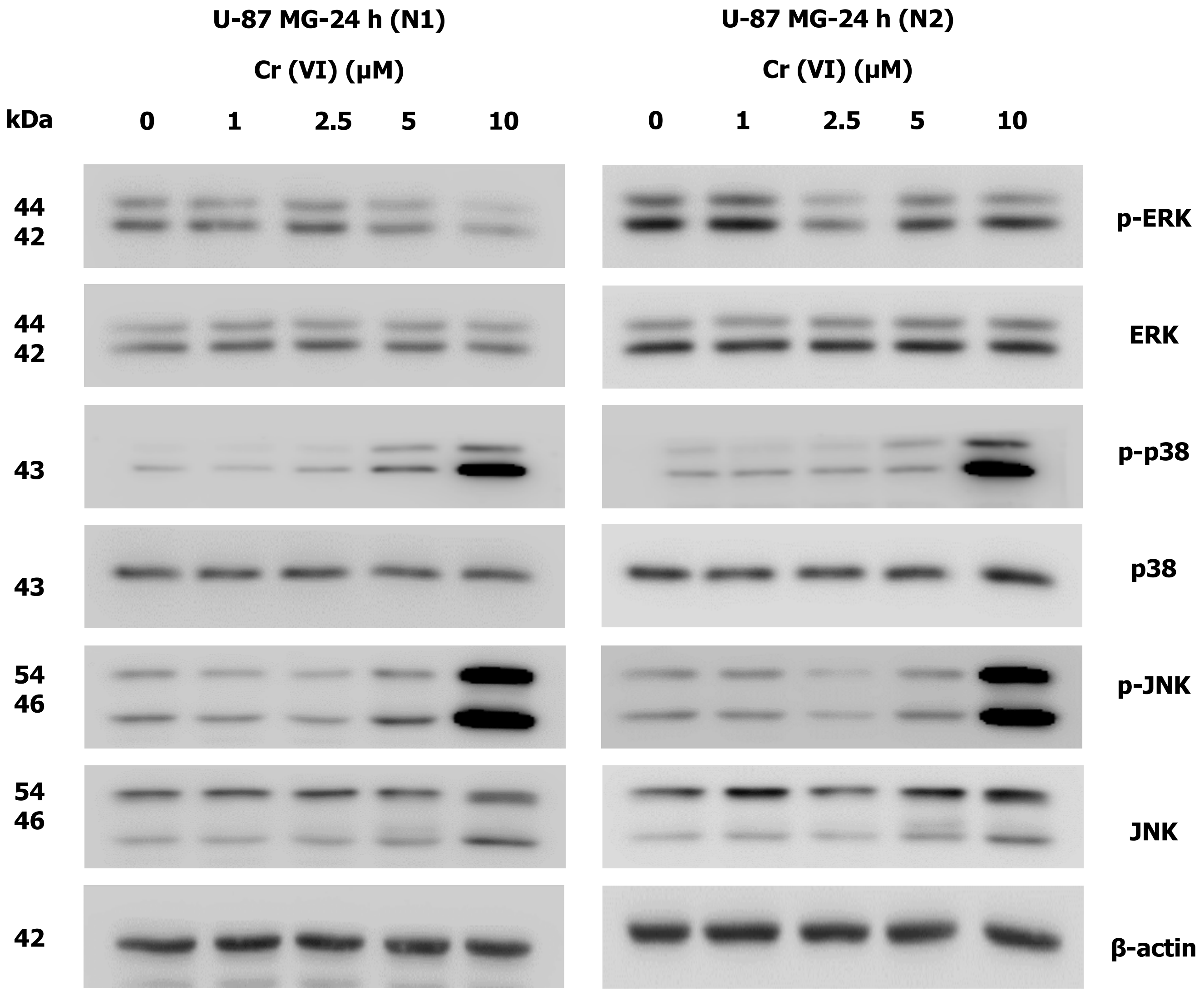

Images of Western Blotting for Figure 7A

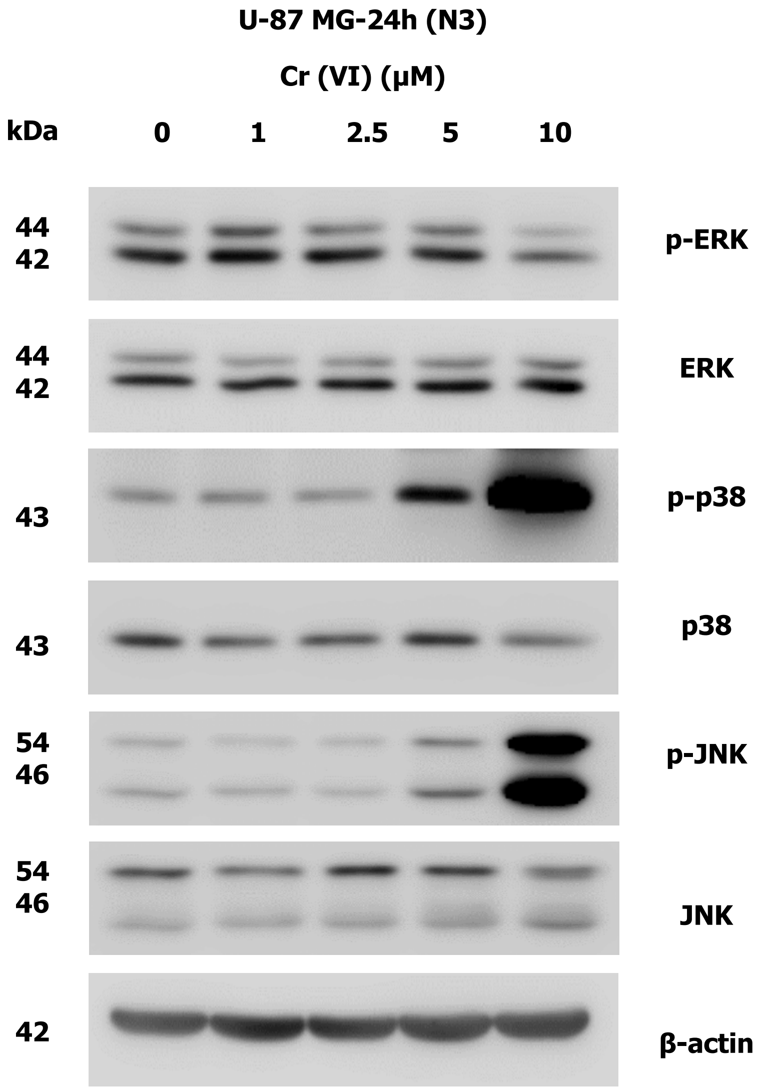

Images of Western Blotting for Figure 8C

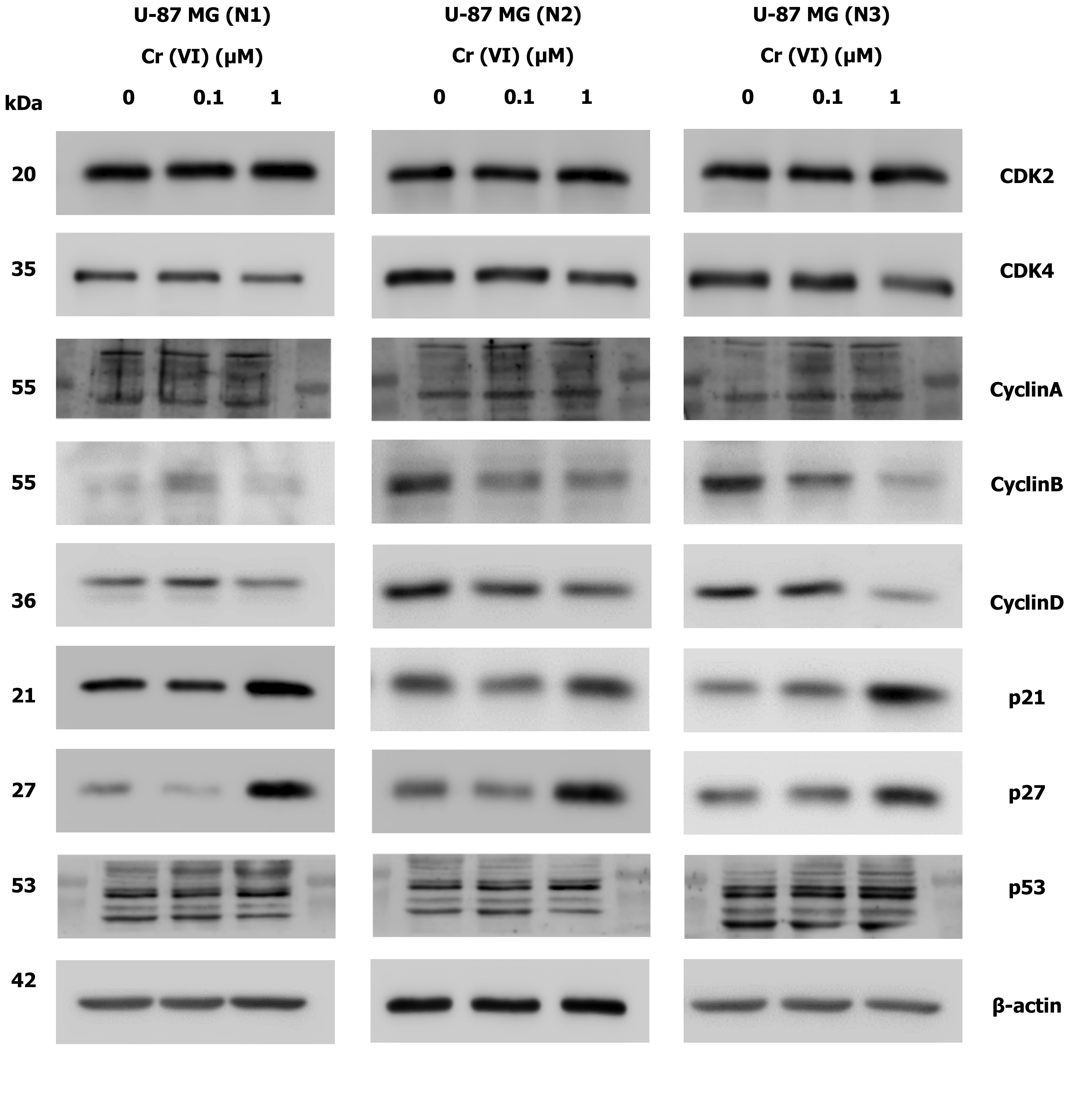

Supplement: Supplementary file 2 — Supplementary Material 2 [file 12011_2026_5046_MOESM2_ESM.pdf]
